# Anatomical and functional evidence for trace amines as unique modulators of locomotor function in the mammalian spinal cord

**DOI:** 10.3389/fncir.2014.00134

**Published:** 2014-11-07

**Authors:** Elizabeth A. Gozal, Brannan E. O'Neill, Michael A. Sawchuk, Hong Zhu, Mallika Halder, Ching-Chieh Chou, Shawn Hochman

**Affiliations:** Physiology Department, Emory UniversityAtlanta, GA, USA

**Keywords:** tyramine, tryptamine, β-phenylethylamine, locomotion, TAAR, dopa decarboxylase

## Abstract

The trace amines (TAs), tryptamine, tyramine, and β-phenylethylamine, are synthesized from precursor amino acids via aromatic-L-amino acid decarboxylase (AADC). We explored their role in the neuromodulation of neonatal rat spinal cord motor circuits. We first showed that the spinal cord contains the substrates for TA biosynthesis (AADC) and for receptor-mediated actions via trace amine-associated receptors (TAARs) 1 and 4. We next examined the actions of the TAs on motor activity using the *in vitro* isolated neonatal rat spinal cord. Tyramine and tryptamine most consistently increased motor activity with prominent direct actions on motoneurons. In the presence of N-methyl-D-aspartate, all applied TAs supported expression of a locomotor-like activity (LLA) that was indistinguishable from that ordinarily observed with serotonin, suggesting that the TAs act on common central pattern generating neurons. The TAs also generated distinctive complex rhythms characterized by episodic bouts of LLA. TA actions on locomotor circuits did not require interaction with descending monoaminergic projections since evoked LLA was maintained following block of all Na^+^-dependent monoamine transporters or the vesicular monoamine transporter. Instead, TA (tryptamine and tyramine) actions depended on intracellular uptake via pentamidine-sensitive Na^+^-independent membrane transporters. Requirement for intracellular transport is consistent with the TAs having much slower LLA onset than serotonin and for activation of intracellular TAARs. To test for endogenous actions following biosynthesis, we increased intracellular amino acid levels with cycloheximide. LLA emerged and included distinctive TA-like episodic bouts. In summary, we provided anatomical and functional evidence of the TAs as an intrinsic spinal monoaminergic modulatory system capable of promoting recruitment of locomotor circuits independent of the descending monoamines. These actions support their known sympathomimetic function.

## Introduction

The classical monoamine neuromodulatory transmitters, dopamine (DA), noradrenaline (NA), and serotonin (5-HT), play an important role in modulating spinal cord sensory, autonomic and motor function (Jacobs and Fornal, 1993; Rekling et al., 2000; Schmidt and Jordan, 2000; Hochman et al., 2001; Millan, 2002; Clarac et al., 2004; Zimmerman et al., 2012; Garcia-Ramirez et al., 2014). Their actions are thought to occur largely via descending monoaminergic neurons that project to the spinal cord (Gerin et al., 1995).

Another group of endogenous monoamines called the trace amines (TAs), include tryptamine, tyramine, and β-phenylethylamine (PEA). The TAs have structural, metabolic, physiologic, and pharmacologic similarities to the classical monoamine transmitters and are synthesized from the same precursor amino acids (Saavedra, 1989). Unlike the classical monoamines, aromatic-L-amino acid decarboxylase (AADC; also called dopa decarboxylase) is the only enzyme required to produce them. Conversion from the TAs to the monoamines does not appear to occur (Berry, 2007).

The TAs have a heterogeneous central nervous system (CNS) distribution with concentrations ranging from 0.1 to 13 ng/g (Durden et al., 1973; Philips et al., 1974a,b, 1975; Boulton, 1976, 1978; Juorio, 1988; Nguyen and Juorio, 1989; Boulton et al., 1990). There are mixed reports on whether the concentrations are higher in spinal cord or brain (Spector et al., 1963; Boulton et al., 1977; Juorio, 1979, 1980; Karoum et al., 1979). While often viewed as metabolic by-products (Boulton, 1976; Berry, 2004), the TAs are clearly neuroactive. For example, in spinal cord PEA enhanced while tyramine depressed monosynaptic reflexes (Kitazawa et al., 1985; Ono et al., 1991). Tyramine also depressed flexion and crossed-extension reflexes (Bowman et al., 1964) consistent with reported antinociceptive actions (Reddy et al., 1980). Tyramine also directly depolarized motoneurons with an EC_50_ comparable to DA (~50 μM) (Kitazawa et al., 1985).

The recent discovery of trace amine-associated receptors (TAARs) establishes a mechanism by which TAs can produce effects of their own (Borowsky et al., 2001; Bunzow et al., 2001). Tyramine and PEA activate TAAR1, while PEA and tryptamine activate TAAR4 (Borowsky et al., 2001). Observations using selective TAAR1 agonists and antagonists and TAAR1 knockout mice demonstrate a clear role for TA actions on CNS TAAR1 receptors. TAAR1 activity appears to depress monoamine transport and limit dopaminergic and serotonergic neuronal firing rates via interactions with presynaptic D_2_ and 5-HT_1A_ autoreceptors, respectively (Wolinsky et al., 2007; Lindemann et al., 2008; Xie and Miller, 2008; Xie et al., 2008; Bradaia et al., 2009; Revel et al., 2011; Leo et al., 2014). This supports a large classical literature on the role of TAs as endogenous neuromodulators of monoaminergic excitability and neurotransmission (Boulton, 1991; Berry, 2004).

TA-induced modulatory actions in the CNS have yet to be linked with specific trace aminergic neuronal systems. Candidate neurons include 16 anatomically segregated collections of D cells (Jaeger et al., 1984). D cells contain the essential synthesis enzyme AADC but no other monoamine synthesis enzymes, and can only synthesize the TAs from their precursor amino acids (Jaeger et al., 1983, 1984; Nagatsu et al., 1988). D1 cells associate with the lumen of the spinal cord central canal (Jaeger et al., 1983). While the function of these neurons is unknown, a morphologically similar population activates locomotor circuits in larval zebrafish (Wyart et al., 2009).

As TAAR1 mRNA is found in the spinal cord (Borowsky et al., 2001), we hypothesize that the TAs and their receptors represent an intrinsic neuromodulatory system. Here, we characterize spinal expression patterns for AADC, tyramine, TAAR1 and TAAR4, then make use of an isolated neonatal rat spinal cord preparation to examine the role of applied TAs on spinal motor circuits (Kiehn, 2006). Overall we show that the TAs can activate complex rhythmic motor behaviors, including locomotor-like activity (LLA), independent of classical monoaminergic mechanisms. Some of these results have been presented in abstract form (Gieseker et al., 2004; Gozal et al., 2006, 2007).

## Materials and methods

All experimental procedures complied with the NIH guidelines for animal care and the Emory Institutional Animal Care and Use Committee. Sprague-Dawley rats aged post-natal day (P) 0–5, P14, and adult were used.

### TAAR PCR

Total RNA was extracted from male and female rat whole spinal cords (P2) using QIAGEN RNeasy Mini Kit (Cat No: 74104). We performed on-column DNase digestion using the same QIAGEN kit. Total RNA was quantified by NanoDrop 2000 (Thermo Scientific). DNase-treated total RNA (1 μg) was retrotranscribed using the iScript cDNA synthesis kit according to the instructions of the manufacturer (Bio-Rad Laboratories, Hercules, CA, USA). Platinum PCR SuperMix was used for PCR reactions following the instructions of the manufacturer (Invitrogen, Cat No: 11306-016). PCR was conducted with the following cycling program: 2 min at 95°C of initial denaturation, followed by 37 cycles of denaturation at 95°C for 30 s, annealing at 60°C for all TAAR primers for 30 s (except TAAR7 and TAAR9 which were at 55°C), and extension at 72°C for 30 s. PCR products were size-separated by electrophoresis in ethidium bromide-stained 2% agarose gels with bands visualized by ultraviolet transillumination.

Primer sequence information for TAARs 1–6, 8a, and 9 were obtained from Chiellini et al. (2007). We also used one of introns of *Rattus norvegicus* strain BN/SsNHsdMCW chromosome 1 (RGSC_v3.4) as a negative control to make sure that the total RNA from the whole spinal cord was not contaminated by the genomic DNA. Primer sequences for GAPDH and RGSC_v3.4 are: GAPDH (NM017008): forward primer, 5′-GCAACTCCCATTCTTCCACCTTTGA-3′; reverse primer, 5′-TTGGAGGCCATGTAGGCCATGA-3′ (139 bp). RGSC_v3.4 intron (NC_005100.2): forward primer, 5′-AGAGTGGTCTGTTGCAAGTGGTCT-3′; reverse primer, 5′-AAGGGTCTCCAGAAACACCCAAGT (723 bp).

#### In situ hybridization

Complete rat spinal cords were dissected out and the whole cords were stored in RNAlater (Qiagen, Valencia, CA) at −80° until use. Total RNA was extracted by using Qiagen RNeasy Mini kits (Qiagen, Valencia, CA). Five micrograms of total RNA was subject to cDNA synthesis with oligo-dT15 primer and SuperScript II Reverse transcriptase (Invitrogen, Carlsbad, CA) for 1 h at 42°C. The reverse transcriptase was inactivated, and RNA was degraded by heating at 95°C for 5 min. Of the 20 μl of cDNA obtained from the synthesis reaction 5 μl were directly added to the PCR reaction using a PCR Mastermix kit (Eppendorf, Hamburg, Germany) containing 1 μM gene-specific primers. The primer used in this study was designed by the Invitrogen-OligoPerfect™ Designer program (Invitrogen, Carlsbad, CA). Non-radioactive single-stranded digoxigenin cRNA probes were used for *in situ* hybridization using methodology reported previously (Zhu et al., 2007). Briefly, single stranded, digoxigenin-labeled antisense and sense probes are transcribed *in vitro* using T7 and Sp6 RNA polymerase (Promega). The probe sequence for AADC is 523–927 bp (GenBank U31884), 404 bp product. The probe sequence used for TAAR1 is 400 bp long (GenBank AF380186). Hybridization was carried out at 68°C overnight with 3 μg/ml digoxigenin-labeled antisense cRNA probe. Sense probes were used at identical concentrations and development reaction as a negative control. Sections were washed with concentrated standard saline citrate (and then incubated with anti-digoxigenin-AP Fab fragments (1:5000, Roche) in blocking buffer overnight at 4°C. The color development reaction was carried out in the dark and neutralized with color stop buffer (10 mM Tris, pH 5, 1 mM EDTA). Slides were then dehydrated through a series of alcohol washes, coverslipped with Vectamount (Vector Labs) and images were captured on a Nikon E800 light microscope (Nikon ACT-1 software).

#### Immunohistochemistry

Sprague-Dawley rats were anesthetized with urethane (1.5 mg/kg), perfused with 1:3 volume/body weight of prefix (0.9%NaCl, 0.1%NaNO_2_, 1 units/1 ml heparin) followed by equal volume/body weight of Lana's fixative (4% paraformaldehyde, 0.2% picric acid, 0.16 M PO_3_); pH 6.9. In a small subset of experiments, P25 rats with and without a transection were used. In many of the experiments, Fluorogold, which does not cross the blood brain barrier, was injected intraperitoneally 24 h prior to sacrifice to retrogradely label most spinal motoneurons (Ambalavanar and Morris, 1989; Merchenthaler, 1991). The spinal cords were then isolated and post-fixed for 1 h in Lana's fixative then cryoprotected in 10% sucrose, 0.1 M PO_3_ until sectioned into 10 um thick sections on a cryostat and processed for immunohistochemistry. All incubations and washes were performed in 0.1 M PO_3_-buffered saline containing 0.3% triton X-100 (PBS-T). Tissue was washed overnight in PBS-T at 4°C followed by incubation in primary antibody for 48–72 h. Slides were then washed three times for 30 min and incubated in secondary antibody. The concentrations for the antibodies can be found in Table 1. In all experiments, omission controls were used for the primary antibodies. In addition, tyramine, TAAR1, and TAAR4 pre-absorption controls were performed with used antibody concentrations (1:100 and 1:1000) absorbed with 20 μg/100 μl of antigen (from respective antibody suppliers) for 1 h prior to incubation. As reported by the supplier (Chemicon) the tyramine antibody has >50,000-fold selectivity over its precursor amino acid (tyrosine), >40,000 selectivity over dopamine, and 800-fold selectivity over the trace amine octopamine. Images were photographed with a Nikon (Tokyo, Japan) digital camera through a Nikon E800 microscope or using an Olympus FV1000 inverted confocal microscope. Images were processed using Corel Draw (Corel, Ottawa, Ontario, Canada).

**Table 1:** **Antibodies used for immunohistochemistry expression**.

**Primary antibody**	**Secondary antibody**	**Tertiary**
Rabbit anti-tyramine 1:1000 (Chemicon) Rabbit anti-TAAR1 1:1000 (Lifespan Biosciences)	Biotin anti-rabbit 1:250 (Jackson Immunoresearch)	Extravidin Cy3 1:1000 (Sigma)
Rabbit anti-DDC 1:00 (Biomol Sciences) Rabbit anti-tyramine 1:100 or 1:1000 (Chemicon) Rabbit anti-TAAR1 1:1000 (Lifespan Biosciences)	cy3 anti-rabbit 1:250 (Jackson Immunoresearch)	
Sheep anti-DDC 1:100 (Biomol Sciences)	FITC anti-sheep 1:100 (Jackson Immunoresearch)	
Goat anti-TAAR4 1:100 (Santa Cruz Biotechnology)	cy3 anti-goat 1:250 (Jackson Immunoresearch)	
Mouse anti-NeuN 1:50 (Chemicon)	FITC anti-mouse 1:100 (Jackson Immunoresearch)	

#### Lipophilic dye labeling

A P7 rat was anesthetized with isoflurane via inhalation for thoracic cord transection. Following dorsal laminectomy to expose lower-thoracic segments of the cord, one section of the cord between T8 and T12 was removed using surgical microdissection scissors. One week later the cord was isolated and preserved in 2% paraformaldehyde fixative, then suspended in agarose gel and labeled with the carbocyanine dye DiI. Crystals of DiI were placed at the cut surface of ventral funiculi. The dye was allowed to diffuse to identify gray matter projections sites.

#### Electrophysiology

Sprague-Dawley rats P0-5 were decapitated, eviscerated, and placed in a bath containing oxygenated (95% O_2_, 5% CO_2_) artificial cerebral spinal fluid (aCSF) containing the following (in mM): 128 NaCl, 1.9 KCl, 1.2 KH_2_P0_4_, 26 NaHCO_3_, 2.4 CaCl_2_, 1.3 MgSO_4_, and 10 glucose (pH of 7.4). The spinal cord was exposed by a ventral vertebrectomy and carefully dissected out of the body cavity leaving the dorsal and ventral roots attached. The spinal cord was secured with insect pins to a chamber with Sylgard (Dow) on the bottom. Glass suction electrodes were applied to dorsal and/or ventral roots, after which the preparation was allowed to recover for at least 1 h before experimentation at room temperature. The ventral root electroneurographic activity was amplified (10,000x), band-pass filtered at 10–3000 Hz, and digitized at 5 kHz (Digidata 1321A, 16-bit; Axon Instruments). Data was captured on a computer with the pCLAMP acquisition software (v8-9, Molecular Devices; Union City, CA). Electrophysiological data analysis was performed using pCLAMP analysis software (Clampfit) or software written in-house using MATLAB. Statistical comparisons were made using ANOVA, Student's *t*-test, or paired *t*-test. The means is reported as mean ± SD.

Motor activity was monitored using glass suction electrodes attached to ventral lumbar roots, typically bilaterally to L2 and L5. Changes in levels of motor activity were quantified by first applying a RC high-pass filter at 1 Hz to reduce drift, then calculating the root mean square of a representative ventral root signal, and comparing 100 s periods before application of the TAs and monoamines and during their period of maximal response. Changes were expressed as a percent increase over baseline. L2 ventral root activity primarily indicates activity in flexors, while L5 ventral root activity primarily indicates activity in extensors (Kiehn and Kjaerulff, 1998). LLA was defined as an alternation between right and left L2 ventral roots, with each L2 ventral root alternating with the L5 root on the same side. LLA was analyzed using the in-house MATLAB software, SpinalMOD (Hochman et al., 2012), which calculated the frequency, peak amplitude, and phase, which was calculated using the middle of the burst (Matsushima and Grillner, 1992).

To evoke the reflexes, constant current stimuli were applied to the dorsal roots while motor activity was recorded from ventral lumbar roots, typically L5. Stimulus intensities were 500 μA and durations ranging from 100 to 500 μs. For experiments examining motoneuron activity in the absence of synaptic transmission, reflexes were abolished after switching from regular aCSF to with high Mg^2+^ (6.5 mM)/low Ca^2+^ (0.85 mM) aCSF or zero Ca^2+^ aCSF.

Neurochemicals, which were stored in 10 or 100 mM stock solutions at −20°C, were added to a directly oxygenated static bath (typically 30 mL) with drug equilibration achieved via rapid recirculation using several syringe-based fluid removal/replacement events. Solution exchange was achieved by replacing the bath aCSF at least three times. Neurochemicals were obtained from Sigma-Aldrich (St. Louis, MO). N-methyl-D-aspartate (NMDA) (3–5 μM), 5-HT (50 μM), noradrenaline (50 μM), and dopamine (50 μM) were applied at concentrations comparable to those used previously in neonatal rat (Cazalets et al., 1995; Kiehn and Kjaerulff, 1996; Kjaerulff and Kiehn, 1996; Cowley and Schmidt, 1997; Kremer and Lev, 1997; Kiehn et al., 1999; Sqalli-Houssaini and Cazalets, 2000; Barriere et al., 2004). The following TAs were used: tryptamine (50 μM), tyramine (1–100 μM), and PEA (50–100 μM). TA doses were chosen to match those of the monoamines under the assumption that they have equivalent transporter uptake and degradation following exogenous application. Due to the efficiency of the monoamine transporters, it has been estimated that the actual dose at receptor sites is ~1/30th that applied (Murray et al., 2011). The aromatic amino acids tyrosine, tryptophan, and phenylalanine were applied at doses between 100 and 200 μM. The non-specific 5-HT receptor antagonist methysergide was applied at 1–10 μM. The serotonin (SERT), dopamine (DAT), and noradrenaline (NET) transport inhibitors applied were: citalopram (1 μM; a SERT inhibitor), buproprion (1 μM; a DAT inhibitor), and clomipramine (5 μM; a SERT and NET inhibitor). The vesicular monoamine transport (VMAT) inhibitor, reserpine, was applied at 10 μM. The organic cation transport (OCT) inhibitors, pentamidine was applied at 200 μM. The protein synthesis inhibitor, cycloheximide, was applied at 100 μM.

## Results

### Anatomical substrate for TA actions in the spinal cord

#### AADC and tyramine are widely expressed in the spinal cord

**Figure 1:**
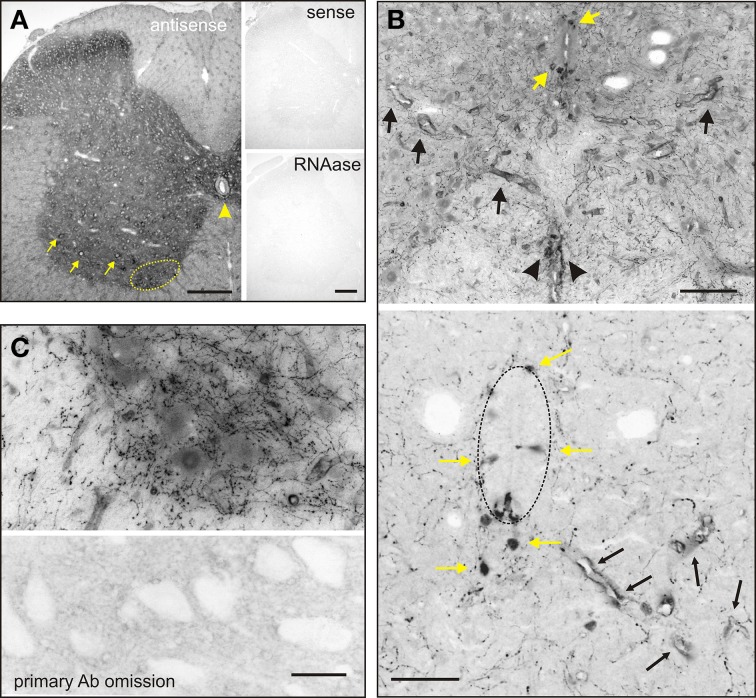
***In situ* hybridization (A) and immunohistochemistry (B,C) reveal AADC labeling throughout the adult spinal gray matter. (A) Left** Low power magnification of AADC antisense *in situ* hybridization. **Right** Sense and RNAase treatment at right confirms specificity of antisense probe. Arrows point to putative motoneurons with perinuclear labeling, and putative D cells below the central canal. **(B) Top** Intense AADC immunolabeling is found in blood vessels (black arrows) and in the D cells (yellow arrows) and in the ventromedial white matter (black arrowheads). Note that some AADC^+^ neurons are also found in the dorsal central canal. **Bottom** Higher power image of central canal region (outlined). Blood vessels are identified with black arrows and AADC^+^ neurons with yellow arrows. **(C)** AADC^+^ terminal arborizations surrounding motoneurons. Most of these arborizations are presumably from descending monoaminergic systems. Bottom panel is a negative control omission of primary antibody. Scale bars: **(A)**, 250 μm; **(B)**, 100 μm top, 50 μm, bottom; **(C)**, 25 μm.

*In situ* hybridization and immunohistochemistry carried out in adult rat lumbar spinal cord showed that AADC was detected with widespread labeling throughout the spinal cord (Figure 1). AADC expression was most notable around the central canal, the ventral funiculus, and in ventral neurons, including motoneurons. There was also some nuclear labeling as seen before (Mann and Bell, 1991). AADC immunolabeling around the central canal included the previously reported D cells (Jaeger et al., 1983; Li et al., 2014). Immunohistochemical staining was undertaken as shown in Figures 1B,C. We observed clear AADC immunolabeling in neurons associated with the central canal, in axonal projections in the ventral funiculus, and in blood vessel endothelia where AADC activity is known to be high (Hardebo et al., 1979; Nagatsu et al., 1988; Li et al., 2014; Pfeil et al., 2014).

**Figure 2:**
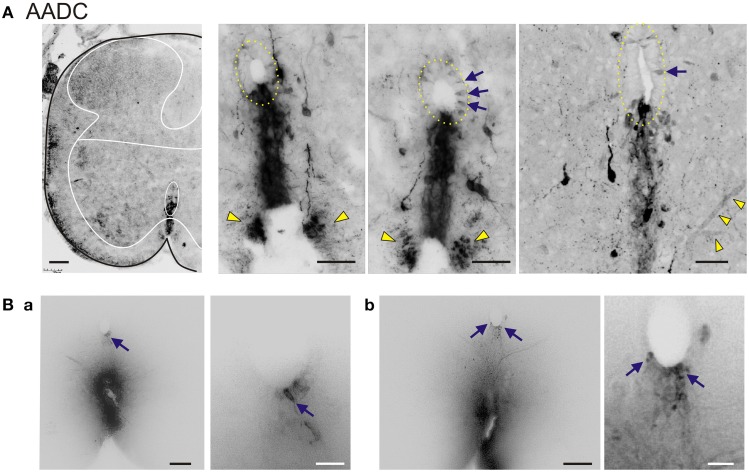
**AADC neuronal subpopulations in the neonatal rat lumbar spinal cord. (A) Left** Low power transverse spinal cord section (10 μm) of AADC immunolabeling. Superimposed is an outline of the spinal cord (black) with interior white lines approximately identifying dorsal and ventral gray matter and central canal region. Note strongest labeling is associated with D cells intermingled with epithelial cells surrounding the central canal (top arrow). Also, note the associated vertical row of cellular labeling projecting ventrally, and bilateral white matter labeling in the ventral funiculus (bottom arrow). There is also weak AADC immunolabeling in putative motoneurons (in lamina IX below dotted lines). **Right** Three panels showing higher power images from separate sections illustrate the diversity of AADC labeling in the ventral medial gray matter region. Epithelial cell layer surrounding central canal is outlined while blue arrows point to example D cells. Common to all is the vertical stream of projections with intermingled cells ventral to the central canal. These appear to end in a white matter tract in the ventral funiculus (yellow arrowheads). Last panel at right is a z-stack of 25, 0.4 μm consecutive images. Note the AADC^+^ blood vessel at bottom right identified by yellow arrowheads. **(B)** Central canal cells project to the ventral funiculus. DiI crystals were placed on fixed lumbar spinal cords at the medial ventral funiculus or just lateral to it and allowed to diffuse to identify gray matter projections sites. Shown are two 70 μm thick transverse sections **(a,b)** from a P14 rat 1 week after with a mid-thoracic spinalization. Images are presented as grayscale with contrast enhancement. Note that when dye placement contacts the midline tract, neurons associated with the central canal are retrogradely labeled. Panels at right are magnified images to highlight retrogradely-labeled neurons in the central canal (corresponding arrows). Scale bars are: **(A)**, 100 μm (left) 50 μm (right three); **(B_a_**,**B_b_)**, 100 and 25 μm for left and right images, respectively.

Next, immunolabeling studies on AADC and tyramine, were conducted in the neonatal spinal cord (Figure 2). This age was chosen to match the age at which the subsequent electrophysiological studies were undertaken. AADC labeling was similar to that found in the adult. Labeled spinal neurons were most notable adjacent and ventral to the central canal, the ventral funiculus, and in ventral neurons, including putative motoneurons (Figure 2A). As observed in the adult, but more strikingly, central canal AADC^+^ neurons projected ventrally in a stream of cells with subsequent termination of presumed axonal projections in the most medial portion of the ventral funiculus (Figure 2A, right). AADC^+^ neurons were also found in the lateral regions near the central canal as well as in the dorsal horn (not shown), although more rarely.

To determine whether cells at the central canal could have axonal projections to the ventral funiculus, DiI crystals were applied to various ventral funicular regions in the fixed lumbar spinal cord of a P14 rat 1 week after thoracic spinalization. When dye placement contacted the midline tract, central canal-associated cells were retrogradely labeled, confirming that cells consistent with the location of D cells can project to the ventral funicular white matter tract (Figure 2B).

**Figure 3:**
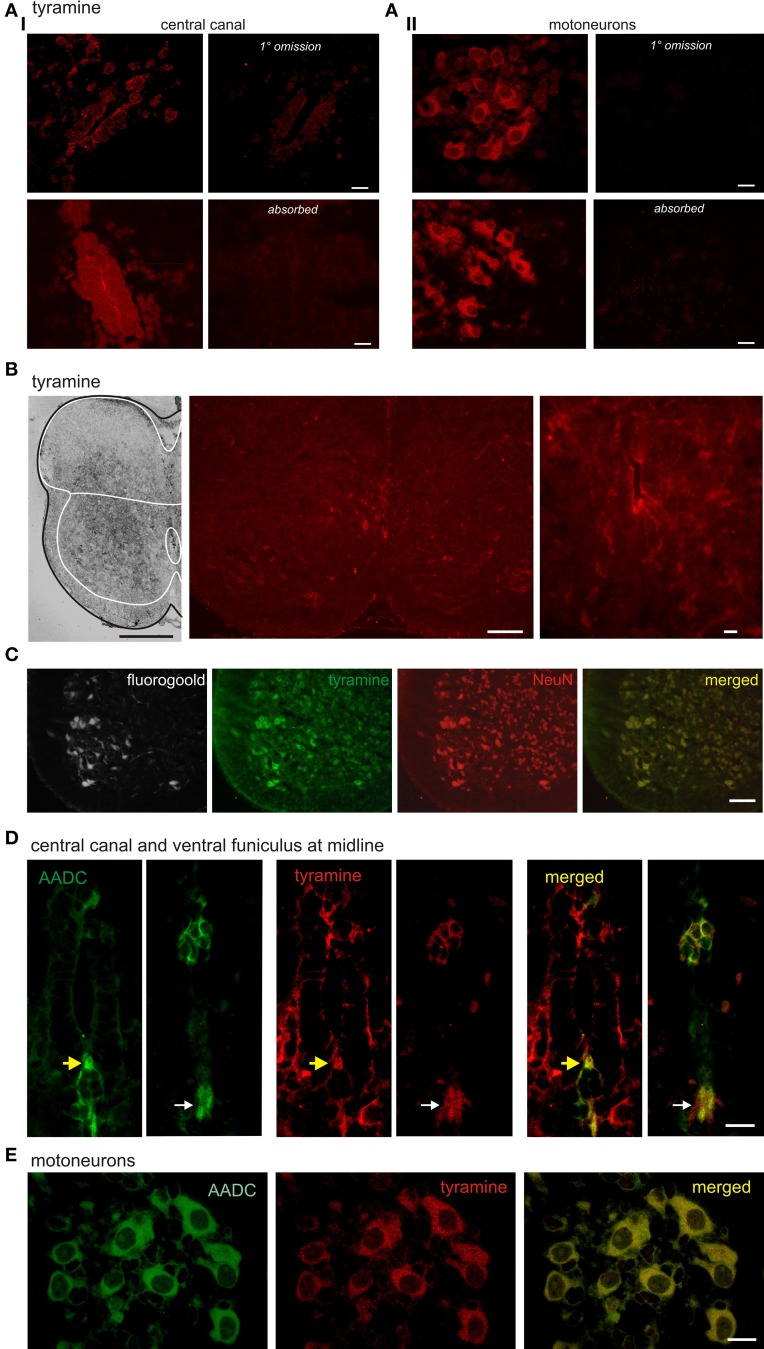
**Tyramine expression in subpopulations of spinal cord neurons and co-expression with AADC. (A)** Tyramine is expressed in cells around the central canal and in putative motoneurons. Fluorescence is minimal following exclusion of primary antibody and greatly reduced in the presence of pre-absorbed antigen. **(B)** Tyramine expression patterns are highly variable. Shown are examples of preferential labeling in ventral gray matter (left) and in association with the central canal (middle and right). **(C)** Preincubation in tyramine leads to widespread neuronal labeling. The isolated spinal cords of P2 rat were pre-incubated in tyramine for 2 h. The day before the treatments, the rat pups were injected with Fluorogold to retrogradely label motoneurons (left). Column 2 shows immunostaining for tyramine. Column 3 provides immunostaining for the neuron-specific marker, NeuN. Column 4 is a merge of the trace amine with NeuN to show that tyramine is observed in many neurons, including motoneurons. **(B)** AADC and tyramine labeling in D cells associated with the central canal (yellow arrows; left panels for each pair) and its ventral cellular projection stream appearing to terminate at a midline tract in the ventral funiculus (right panels in each pair). White arrows identify ventral funiculus. Note D cells, ventral midline cells, and ventral funiculus are co-labeled. **(C)** AADC and tyramine immunolabeling in putative motoneurons. While AADC produces more uniform cytoplasmic labeling (left), tyramine labeling includes larger puncta which are not co-labeled in merged image at right. All images in B and C are high power confocal images with an optical section of 0.4 μm. Scale bars are: **(A)**, 20 μm, **(B)**, 10 μm; **(C)**, 100 μm; **(D,E)**, 20 μm.

Tyramine immunolabeling was characterized by enormous variability between animals, ranging from widespread to near-absent. This variability may reflect the dynamic sensitivity of tyramine to rates of synthesis and degradation. Expression patterns included preferential ventral horn labeling, particularly associated with the central canal and in motoneurons (Figures 3A,B). To increase detectability and further demonstrate that tyramine immunolabeling was neuronal, we preincubated spinal cords in 100 μM tyramine for 2 h. Tyramine labeling co-localized with the neuron specific marker NeuN, including in retrogradely-labeled Fluorogold^+^ motoneurons (Figure 3C).

Tyramine commonly co-expressed with AADC in central canal D cells, in the ventral stream of cells at the midline, and in the ventral funiculus (Figure 3D). These results are consistent with the notion that D cells and related midline neurons synthesize tyramine. We also observed co-labeling in putative spinal motoneurons (Figure 3E).

#### Trace amine-associated receptor expression in the spinal cord

**Figure 4:**
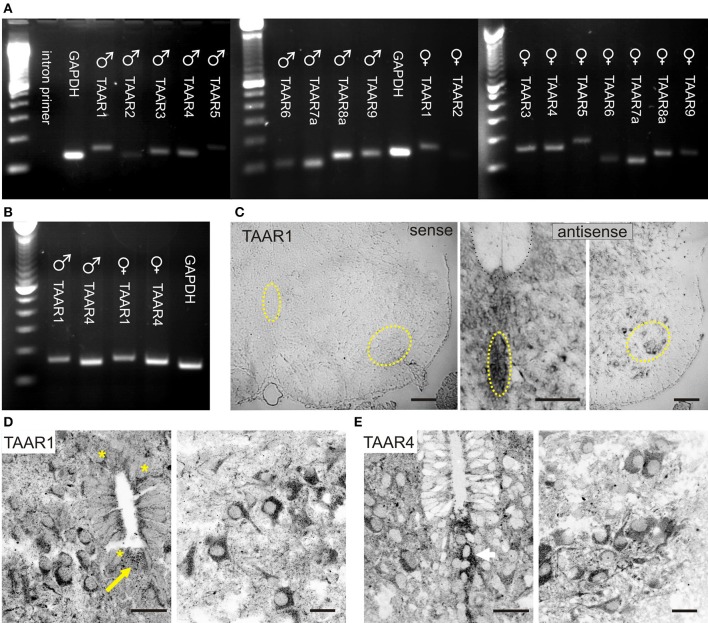
**TAARs are expressed in neonatal rat spinal cord. (A)** RT-PCR evidence of TAAR expression in both sexes. In males, TAARs 2,5, and 6 expression was weakest, and in females, TAARs 2,6, and 9 were weakest. **(B)** RT-PCR series directly comparing TAAR1 and TAAR4 expression in both sexes as these receptors are known to be activated by trace amines. **(C)** TAAR1 mRNA expression in spinal neurons. *In situ* hybridization reveals TAAR1 labeling throughout the P2 rat spinal cord ventral horn (compare sense to antisense). Highlighted regions in sense panel approximately identify same central canal and motor nucleus location in antisense panels. **(D)** TAAR1 receptor immunolabeling is found in many spinal neurons, including those associated with the central canal (asterisks; left), and in the ventral horn, including putative motoneurons (right). Note thread-like projections adjacent to epithelial cells with expansion in central canal interior. **(E)** TAAR4 receptor immunolabeling is also found in many spinal neurons and appears to localize in cells ventral to the central canal (left) and in the gray matter ventral horn, including putative motoneurons (right). All images are grayscale negatives. Scale bars: **(C)**, 100 μm; **(D,E)**, 25 μm. Confocal images in **(D,E)** were taken at 0.30 μm optical section thickness.

Given that 17 TAAR rat genes have been identified, we tested for the presence of TAAR gene expression in the spinal cord. RT-PCR was undertaken on total RNA prepared from neonatal rat spinal cord of both sexes. We observed detectable transcripts for TAARs 1–6, 7a, 8a, and 9 (Figures 4A,B). Because TAAR1 is the only receptor for which there has been detailed study, we also undertook *in situ* hybridization for TAAR1 in the neonate. Widespread spinal cord labeling was observed including around the central canal and in the ventral horn region associated with motoneurons (Figure 4C).

Immunolabeling for both TAAR1 and TAAR4 was found throughout the neonatal spinal cord, including in motoneurons and in neurons around the central canal (Figures 4D,E). TAAR1 and TAAR4 labeling in all neurons appeared intracellular, consistent with previous reported results for TAAR1 (Miller, 2011). A cytoplasmic location of ligand and receptor (e.g., tyramine and TAAR1) supports intracellular activation of signal transduction pathways, as suggested previously (Miller, 2011). The presence of TAAR1 and TAAR4 provides for actions mediated by tyramine and PEA, and tryptamine and PEA, respectively (Borowsky et al., 2001; Bunzow et al., 2001).

### TA actions on motor and locomotor activity *in vitro*

**Figure 5:**
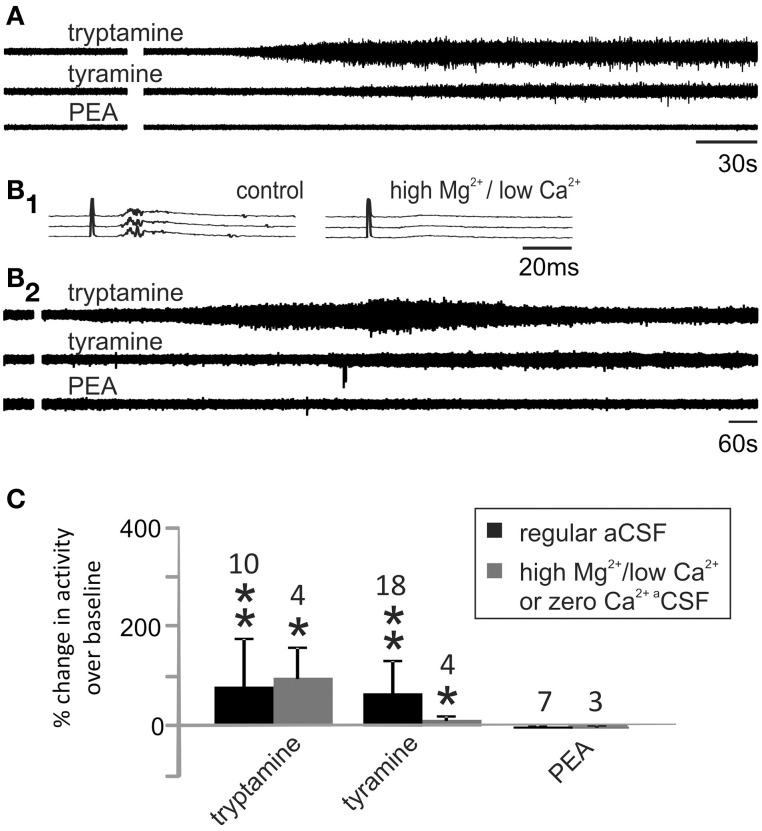
**Tyramine and tryptamine increase motor activity. (A)** Tryptamine and tyramine increased lumbar ventral root activity while PEA was without effect. **(B_1_)** Dorsal root stimulation evoked reflexes (left) were abolished when synaptic transmission was blocked in high Mg^2+^ (6.5 mM), low Ca^2+^ (0.85 mM) containing aCSF (right). **(B_2_)** Under these conditions, direct excitatory actions were still observed for tryptamine and tyramine. **(C)** Changes in ventral root activity were quantified compared to baseline activity levels with and without synaptic transmission (^*^ indicates *p* < 0.05 and ^**^ indicates *p* < 0.01). Samples sizes are listed.

To examine whether tyramine, tryptamine, and PEA increased motor excitability, population motor activity was recorded from a lumbar ventral root before and after their application. As shown in Figure 5A, tryptamine and tyramine increased motor activity while PEA did not. To test whether the TAs acted directly on motoneurons, chemical synaptic transmission was minimized by replacing the regular aCSF with one having high Mg^2+/^ low Ca^2+^ or in nominally zero Ca^2+^ (Shreckengost et al., 2010). Under these conditions, dorsal root stimulation-evoked reflexes were abolished (Figure 5B_**1**_). Subsequently, tryptamine and tyramine still increased motor activity (Figures 5B_**2**_,C).

**Figure 6:**
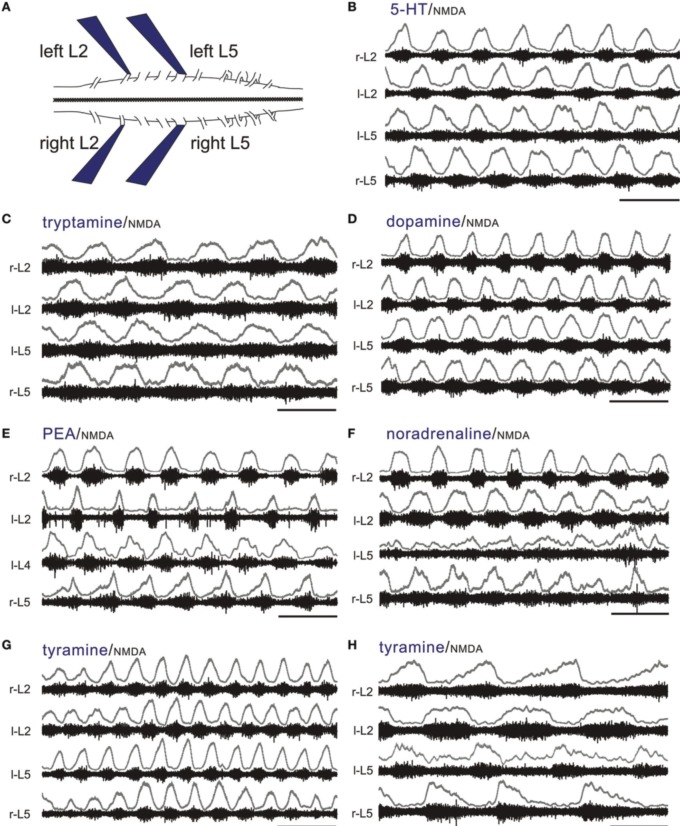
**The TAs and monoamines can all produce a continuous LLA pattern. (A)** Experimental setup (left). All of the electrophysiological experiments use the thoraco-lumbar isolated neonatal rat spinal cord maintained *in vitro*. Suction electrodes are placed on lumbar L2 and L5 ventral roots bilaterally to monitor population motoneuron flexor and extensor activity, respectively. LLA is shown as an alternation between right and left L2 flexors, with each flexor rhythm alternating with the L5 extensor rhythm on the same side. **(B)** Typical locomotor rhythms observed with co-application of 5-HT and NMDA. **(C–G)** Tryptamine, dopamine, PEA, NA, and tyramine can also produce a continuous LLA pattern comparable to 5-HT in the presence of NMDA. **(H)** Tyramine can also produce a slower LLA pattern. The upper traces in gray at each lumbar root represent the rectified/low-pass filtered equivalent of the raw waveforms below them. Scale bars are 10 s in all panels.

**Figure 7:**
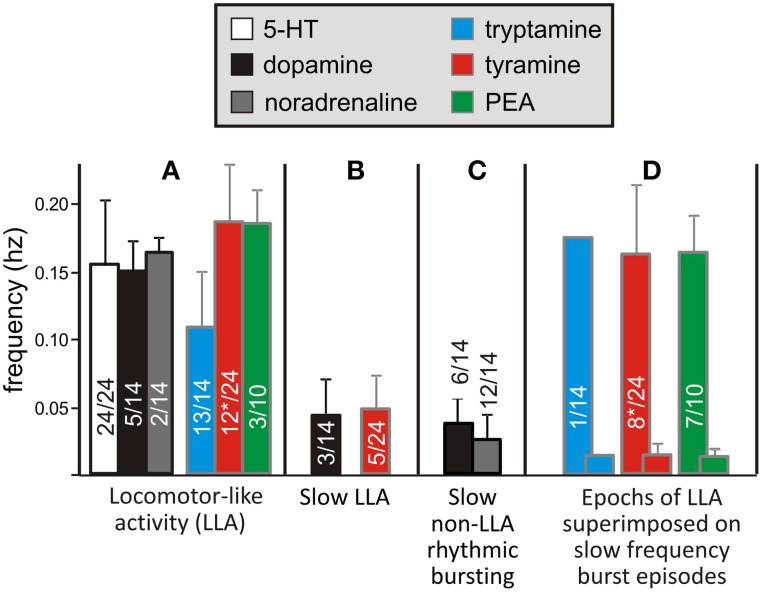
**Similarities and differences in motor burst phenotypes observed with the monoamines and TAs**. Four characteristic categories **(A–D)** describe the range bursting patterns observed. Frequencies varied depending on the type of bursting. **(A)** In the neonatal rat, 5-HT/NMDA-induced LLA has a characteristic frequency range, which was also seen when NMDA was co-applied with the other amines. **(B)** Dopamine and tyramine also produced a much slower rhythm, but with the same flexor-extensor left-right locomotor-like coordination. **(C)** NA and tyramine could also produce a much slower rhythm, but without locomotor-like coordination. **(D)** Seen only with the TAs, this episodic pattern is described as “Bouts of LLA superimposed on slow frequency burst episodes.” LLA and the slower episodic bursts are represented as overlapping bars in this grouping. The frequencies of slow LLA **(B)**, slow non-LLA rhythmic bursting **(C)**, and the slow frequency of episodic bursting **(D)** were statistically slower than LLA. Asterisks (^*^) denote inclusion of an animal for tyramine in two categories as both behaviors emerged at different times.

NMDA is commonly co-applied with 5-HT to produce a stable locomotor-like rhythm in the isolated spinal cord maintained *in vitro* with a coordination comparable to normal locomotion when limbs remain attached (Sqalli-Houssaini et al., 1993; Kjaerulff and Kiehn, 1996; Hayes et al., 2009). This LLA can be monitored from ventral roots as left/right and ipsilateral alternation of bursts between flexors (L2) and extensors (L5) (Figure 6A) (Kiehn and Kjaerulff, 1998). To test for locomotor promoting actions of the TAs and classical monoamines, we co-applied NMDA at concentrations that never produced stable LLA on its own (3–5 μM; *n* = 15). Tryptamine (*n* = 14/19), tyramine (*n* = 24/26), PEA (*n* = 10/10), NA (*n* = 14/14), DA (*n* = 14/14), and 5-HT (*n* = 24/24) reliably recruited rhythmic motor activity. Of those expressing rhythmic motor activity, a locomotor-like rhythm comparable to 5-HT was observed for DA, NA and the TAs with the following incidences: DA (5/14), NA (2/14), tryptamine (13/14), tyramine (12/24), and PEA (3/10). Examples are shown in Figures 6B–G. In these instances, all amines led to bursting at frequencies statistically indistinguishable from 5-HT (Figure 7A). Since the bursting patterns produced by the TAs are often similar to 5-HT, the TAs likely recruit the same pattern-generating circuits. Tyramine (*n* = 5/24; Figure 6H) and dopamine (*n* = 3/14) also produced a second rhythm that was significantly slower, but with comparable locomotor-like coordination (Figure 7B). DA (*n* = 9/14; 0.04 ± 0.02 Hz) and NA (*n* = 12/14; 0.02 ± 0.02 Hz) also produced rhythmic patterns of continuous bursting that were non-locomotor-like in coordination and significantly slower than 5-HT-evoked rhythms (Figure 7C).

**Figure 8:**
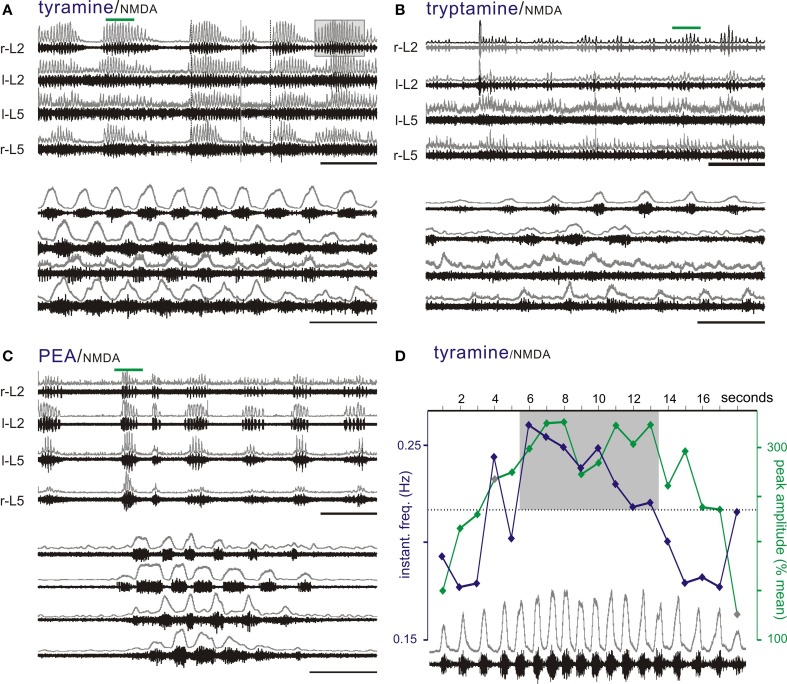
**The TAs produce episodic bursting patterns that are different than the regular pattern seen with 5-HT in the presence of NMDA. (A–C)** Tyramine, tryptamine, and PEA can produce episodic rhythmic motor bursting patterns with bouts of locomotor-like bursting interrupted by quiescent periods. Bottom panels are expanded time scales of time periods identified by green bars to show in detail the locomotor-like coordination. Typically, episodic bursts are concurrent on all ventral roots. **(D)** Episodic bouts of locomotor-like activity are frequently associated with wax and wane changes in amplitude and frequency. Shown is the bout highlighted by the box on r-L2 in panel A with overlaid plots of instantaneous frequency (blue) and peak amplitude of the rectified response (green). Note the trend for amplitude and frequency to increase then decrease over the episodic bout of locomotion. Events within the shaded box emphasize the dominance of higher frequency/higher amplitude values in the middle of the episode of bursting. The upper traces in gray at each lumbar root represent the rectified/low-pass filtered equivalent of the raw waveforms below them. Scale bars are 100 s in top panels and 10 s in bottom panels.

Interestingly, tryptamine (*n* = 1/14), tyramine (*n* = 8/24), and PEA (*n* = 7/10) were able to generate a bursting pattern never seen with the monoamines in this preparation. The pattern was more complex with episodic bouts of bursting (Figure 7D). During these bouts, burst frequencies were statistically indistinguishable from 5-HT locomotion. Episodic bouts of bursting were variable but usually concurrent across roots and alternated with comparatively quiescent periods at very slow average frequencies (Figure 8). For tyramine, bursting patterns interconverted between continuous locomotion to episodic bouts in one experiment. Notably, bouts of episodic bursting were the dominant activity pattern observed for PEA (Figure 7C). Expressed bouts of episodic bursting were highly variable, containing up to 47 events within an episode and with quiescent periods varying from 5 to 230 s.

Frequently, there were progressive increases and decreases in the locomotor burst amplitude within each episode, which were noticeable in at least some bouts for all TAs tested (Figure 8). Similarly, locomotor frequency could be seen to wax and wane during these bouts, supporting a sinusoidal drive to the CPG. This is highlighted in Figure 8A with progressive increases then decreases in burst amplitude, and locomotor frequency are further quantified in Figure 8D.

### Evidence that the TAs act at unique binding sites

#### Differences in receptor pharmacology

5-HT is thought to activate spinal locomotor circuits via 5-HT_2_ and 5-HT_7_ receptors (Madriaga et al., 2004; Liu and Jordan, 2005; Liu et al., 2009). Methysergide is a high affinity non-selective 5-HT_1_, 5-HT_2_, and 5-HT_7_ receptor antagonist (Madriaga et al., 2004) and a tryptamine binding site antagonist (Martin et al., 1988). To determine if the TAs and other monoamines had a similar sensitivity to methysergide as 5-HT, methysergide was added to TA and monoamine–induced rhythms (with NMDA) at progressively increasing doses (Table 2). Tryptamine was more sensitive than 5-HT to methysergide block. Tyramine was variably sensitive to methysergide block, while PEA, NA, and DA-evoked LLA was methysergide-insensitive even at the highest dose tested (10 μM). Based on methysergide sensitivity, it appears likely that tyramine and PEA actions are not dependent on 5-HT receptor activation. Methysergide block of tryptamine-evoked LLA may be via competitive block at 5-HT receptors and/or at the methysergide-sensitive tryptamine binding site (Martin et al., 1988).

**Table 2:** **Methysergide preferentially blocks tryptamine and 5-HT-induced locomotor like activity**.

**Drug**	**Methysergide dose**			
	**1 μM**	**2 μM**	**5 μM**	**10 μM**
5-HT	2/8	5/5		
Tryptamine	6/6			
Tyramine	1/6	0/5	2/5	0/3
PEA	0/3	0/3	0/3	0/3
Noradrenaline	0/4	0/4	0/4	0/4
Dopamine	0/6	0/4	0/4	0/4

*Following induced LLA, methysergide was applied in progressively increasing doses as shown to assess differential monoamines agonist sensitivity to block*.

#### Temporal differences in activation onset

**Figure 9:**
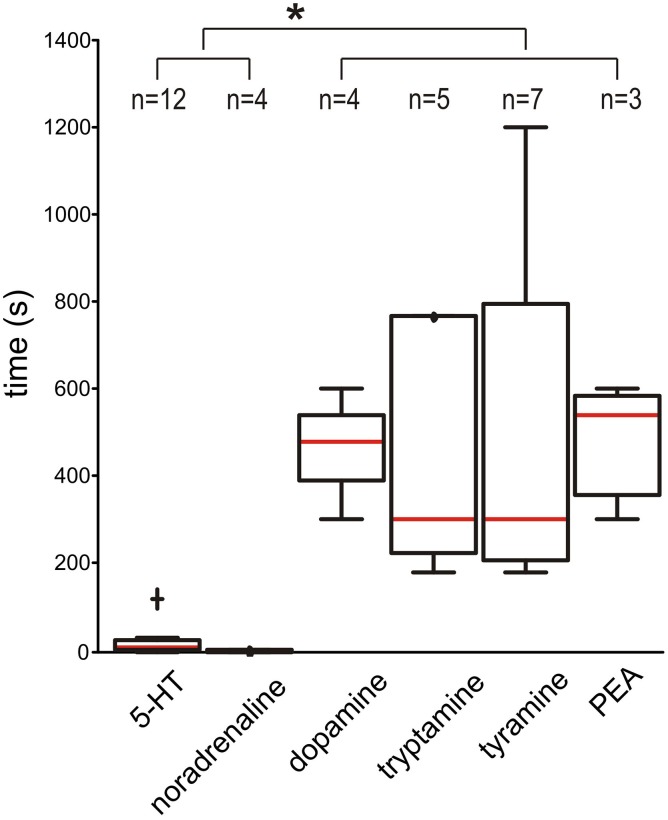
**Dopamine and the TAs take a significantly longer time to initiate bursting than 5-HT and NA**. The box-and-whisker plots show the time it takes to initiate bursting (always in the presence of NMDA). The red line is the median and individual outliers, defined as more than 1.5 times the interquartile range were observed for 5-HT (cross) and for tryptamine at 1800 s (not shown). Asterisk (^*^) indicates *p* < 0.05. Samples sizes are shown at top.

When time to burst onset after bath application was examined, 5-HT and NA had rapid burst onset (medians of 7.5 and 2.5 s, respectively). In comparison, burst onset took dramatically longer for DA, tryptamine, tyramine, and PEA (medians of 480, 300, 300, and 540 s, respectively; all are *p* < 0.05) (Figure 9). This suggests that the TAs and DA are not acting predominantly on plasma membrane metabotropic receptors.

#### Possible sites of action

**Figure 10:**
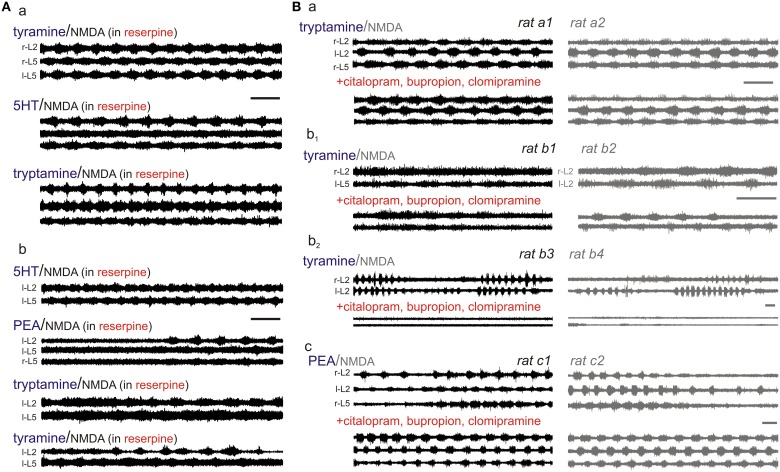
**TA-induced LLA is not principally via uptake by high-affinity plasma membrane or vesicular monoamine transporters. (A)** The vesicular monoamine transport inhibitor, reserpine (10 μM), did not prevent TA-induced modulatory actions. Shown is evoked LLA in two rats **(a,b) (B)** TA-evoked actions were tested before and after combined application of the transport inhibitors, citalopram (1 μM; a SERT inhibitor), buproprion (1 μM; a DAT inhibitor), and clomipramine (5 μM; a SERT and NET inhibitor). The transport inhibitors were usually without effect as shown for tryptamine **(a)**, but could also depress (**b**; in this case tyramine) or facilitate LLA (**c**; in this case PEA). In all panels, 5-HT and the TAs were always in the presence of NMDA. Scale bars are 10 s in all panels.

***TA-evoked locomotor-like activity is independent of the vesicular monoamine transporter***. To test whether TA-evoked actions required interactions with the vesicular monoamine transporter (VMAT), we compared the locomotor-inducing ability of the TAs to those of 5-HT (always in the presence of NMDA) in bath chambers pre-incubated with the VMAT inhibitor reserpine (>40 min) (10 μM; Figure 10A). In the presence of reserpine, 5-HT and tryptamine evoked LLA in all cords tested (*n* = 5/5). In comparison, tyramine- and PEA-evoked LLA was seen in 4.5 and 3/5 cords, respectively. As reserpine has moderate binding affinity to D_2_ and D_3_ dopamine receptors (Toll et al., 1998) and also interferes with DAT and NET (Yamamoto et al., 2007; Mandela et al., 2010), non-specific actions including at a PEA binding site may have accounted for the reduced incidence of LLA with PEA. Overall, the VMAT inhibitor, reserpine, does not prevent the expression of TA-evoked LLA.

***TA-evoked locomotor-like activity is independent of high-affinity monoamine transporters located on descending monoaminergic terminals***. The high-affinity monoamine transporters for 5-HT (SERT), DA (DAT), and NA (NET) control the extracellular concentrations of monoamines and maintain presynaptic function (Torres et al., 2003). Since the monoamine transporters are also a substrate for transport of TAs (Xie et al., 2007), a mechanism by which the TAs could exert their action is by transporter-mediated presynaptic uptake at descending monoaminergic terminals. For example, the TAs could elevate extracellular monoamines by competing for uptake or displacing biologically active biogenic amines from their storage leading to efflux via reverse transport (Premont et al., 2001). Additionally, once transported intracellularly, they could act on presynaptic TAARs to alter basal activity (Miller, 2011).

To explore this, we compared the motor rhythm-inducing actions of the TAs or monoamines to those seen in the presence of co-applied SERT, DAT, and NET transport inhibitors (citalopram, 1 μM; bupropion, 1 μM; and clomipramine, 5 μM, respectively). Given the variability in expression of TA-evoked responses observed in earlier experiments, we undertook most subsequent studies using littermate pairs of spinal cords in the same experimental chamber (Figure 10B). 5-HT-evoked LLA was not significantly altered (*n* = 12; 0.16 ± 0.06 Hz without vs. 0.15 ± 0.04 Hz with inhibitors). Similarly, PEA-evoked LLA was unaffected by the transport inhibitors in 9 animals (0.17 ± 0.09 Hz vs. 0.15 ± 0.06 Hz). Tryptamine-induced motor rhythms were comparable in 7/8 animals and blocked in another (0.11 ± 0.03 Hz without vs. 0.08 ± 0.06 Hz with inhibitors). Tyramine-evoked actions were blocked in 2/11 animals. Interestingly, tyramine block occurred in both spinal cords of a single chamber, littermate-paired experiment (Figure 10Bb_**2**_). In the remaining 9 neonates, LLA was not blocked (0.08 ± 0.07 Hz before vs. 0.12 ± 0.10 Hz in the presence of inhibitors). Thus, the TAs can activate motor rhythms independent of actions on high-affinity monoamine transporters in the isolated spinal cord. As no spinal neurons express NET, SERT, or DAT, direct actions on spinal circuits are strongly implicated.

***Intracellular transport of tyramine and tryptamine are required for locomotor-like activity: dependence on low affinity Na^+^-independent membrane transporters***. OCTs belong to a family of Na^+^ and Cl^−^ independent bidirectional solute carrier (SLC) transporters, SLC22A (Jonker and Schinkel, 2004). The family contains three subtypes (OCT1-3) and all appear widely expressed in the spinal cord (Allen_Spinal_Cord_Atlas, 2009). Tyramine was shown to be the best physiological substrate for OCT1 and OCT2 (Schomig et al., 2006). In comparison, DA, NA, and 5-HT clearance was too low for a primary role in transport while tryptamine and PEA actions were not tested. The plasma membrane monoamine transporter (PMAT) is another at Na^+^-independent transporter with comparable pharmacological and transport properties to the OCTs. PMAT transports biogenic amines including tyramine and tryptamine (Engel and Wang, 2005) while PEA acts as a potent cis-transport inhibitor (Ho et al., 2011). PMAT has widespread expression in neurons in brain and spinal cord (Engel and Wang, 2005; Allen_Spinal_Cord_Atlas, 2009).

**Figure 11:**
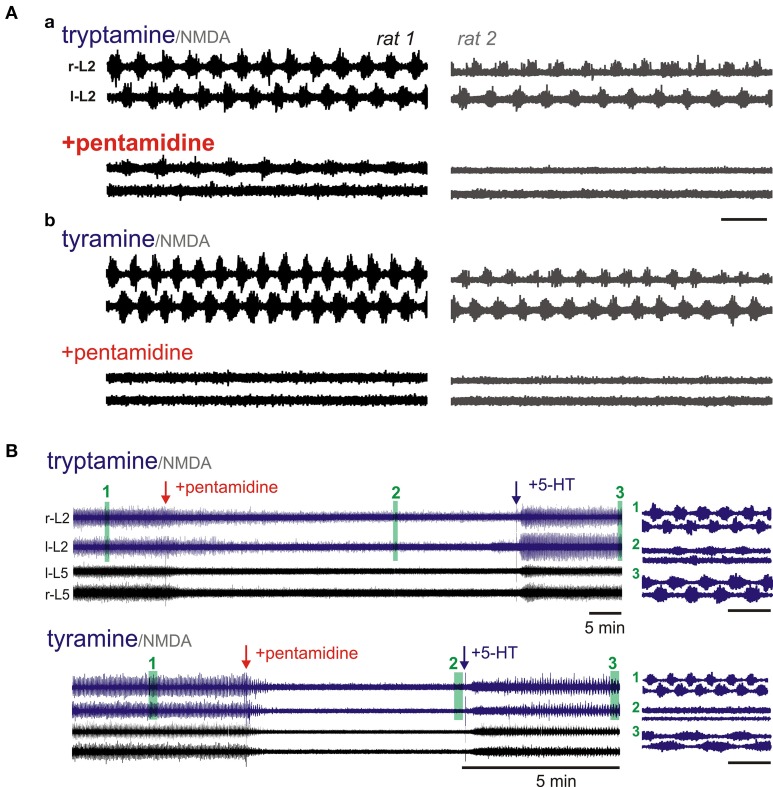
**OCT/PMAT transport inhibitors depress or block tryptamine and tyramine-induced LLA**. Shown are simultaneous recordings from littermates in the same chamber. **(A_a_)** Pentamidine (200 μM) depressed tryptamine-induced LLA. **(A_b_)** Following wash, tyramine-induced LLA was also depressed by pentamidine. In both **(A_a_,A_b_)**, LLA re-emerged with subsequent addition of 5-HT (not shown). **(B)** Time course of pentamidine-induced depression of tyramine and tryptamine-induced LLA in the same animal. LLA re-emerges after application of 5-HT (50 μM). Arrows denote time of pentamidine and 5-HT applications. R-L2 and l-L2 recordings at right present epochs with a shorter time scale for the periods highlighted by numbered green bars. Scale bars are 10 s unless otherwise shown.

Pentamidine is an OCT inhibitor with IC_50_ values of 0.4 and 5.1 μM for OCT1 and OCT2, respectively (Jung et al., 2008). The effects of pentamidine were only tested on tyramine and tryptamine-evoked LLA (Figure 11A). Tyramine-induced LLA (0.19 ± 0.02 Hz) was blocked in all 8/8 cords tested (*p* < 0.001). Tryptamine was also tested in 5 of these animals. Pentamidine completely blocked tryptamine-induced LLA in 2/5 animals with reduced overall frequency in the other 3 animals (0.16 ± 0.02 to 0.07 ± 0.07 Hz; *p* < 0.05). Depressant actions were associated with dramatic reductions in the magnitude of observed activity (Figure 11B). When 5-HT was subsequently applied, LLA re-emerged in all 5 animals (0.17 ± 0.08 Hz) supporting an independence of pentamidine actions on 5-HT-evoked LLA (Figure 11B). It is noteworthy that pentamidine is also a NMDA receptor non-competitive antagonist, but did not prevent expression of 5-HT/NMDA LLA (Reynolds and Aizenman, 1992; Reynolds et al., 1993).

***Evidence that the TAs can be endogenously synthesized from their aromatic amino acid precursors to generate locomotor-like activity***. Intrinsic spinal cord AADC activity should enable endogenous synthesis of the TAs from their precursor aromatic amino acids (AAAs), phenylalanine (for PEA), tyrosine (for tyramine), and tryptophan (for tryptamine). We therefore tested whether bath application of AAAs lead to the expression of rhythmic motor activity. Tyrosine (*n* = 5), tryptophan (*n* = 3), or phenylalanine (*n* = 3) were applied at doses between 100 and 200 μM with or without NMDA. In two additional experiments, the AAAs were co-applied. In all cases, no obvious maintained motor rhythms were observed (not shown).

As *in vitro* experiments are undertaken in the absence of essential amino acid containing media, we explored whether the lack of observed effect was related to lack of substrate availability. In the developing neonate, available cytoplasmic AAAs may be preferentially directed toward protein synthesis. We therefore tested the effects of complete arrest of protein synthesis using cycloheximide (100 μM). Cycloheximide is known to increase intracellular levels of AAA (Beugnet et al., 2003) and inhibition is mostly reversible within 1 h (Abbas, 2013).

**Figure 12:**
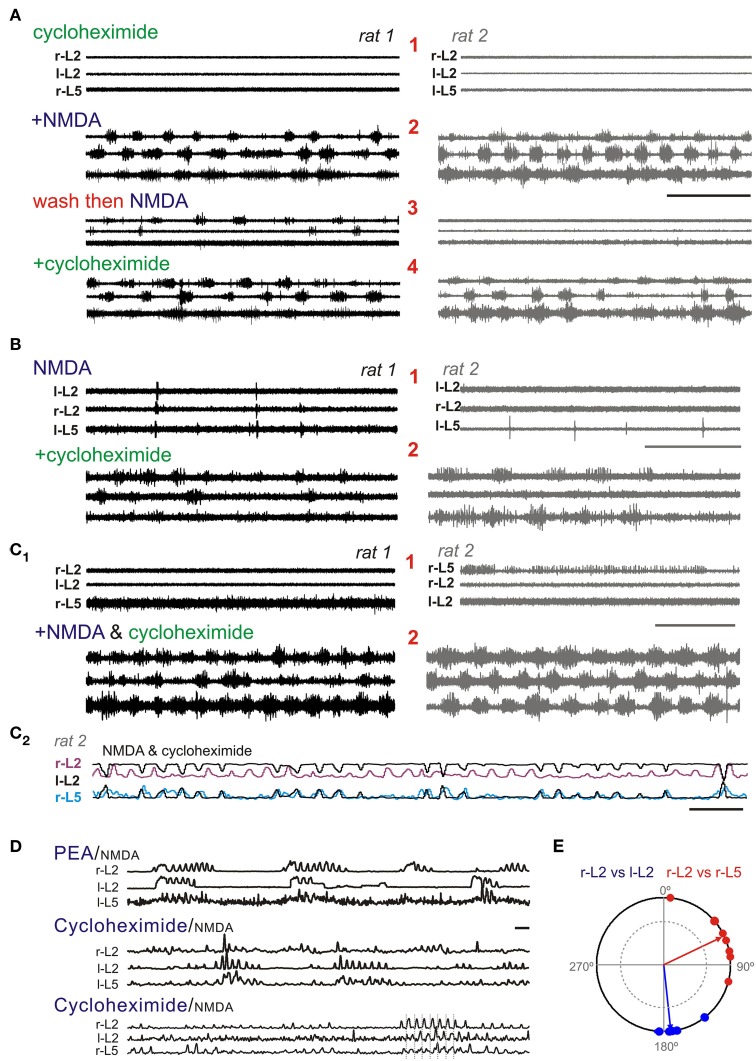
**In the presence of NMDA, cycloheximide-induced accumulation of intracellular amino acids leads to the expression or facilitation of LLA**. Shown are three separate experiments with pairs of littermate matched spinal cords in the same recording chamber. **(A)** Following pre-incubation of cycloheximide **(1)**, applied NMDA leads to the expression of LLA in both cords **(2)**. Replacement of solution and subsequent application of NMDA **(3)** reinstates some bursting in cord, but a full LLA pattern emerges in both cords only after reapplication of cycloheximide **(4)**. **(B)** NMDA generated some spiking in both cords of this pair **(1)** while addition of cycloheximide led to LLA in both pairs. **(C_1_)** In this pair, some spontaneous excitability was seen initially (1) and NMDA^+^ cycloheximide led to coordinated LLA **(2)**. Note the overall similarity in response in paired spinal cords. **(C_2_)** Low-pass filtered recording of additional recorded LLA activity in *rat 2* after cycloheximide and NMDA. L-L2 activity (black) is inverted and adjacent to r-L2 to show anti-phase left-right coordination between flexors, and overlapped with r-L5 activity to show phase coupling with contralateral extensor activity. **(D)** Comparison to PEA evoked rhythm. Longer duration recordings with rectified, low-pass filtered trace compare PEA expression pattern to cycloheximide/NMDA LLA in two separate animals. Scale bar is 10 s in all **(A–D)**. **(E)** Phase relationship of r-L2 burst onset (from 0°) to l-L2 and r-L5 burst onsets (blue and red symbols, respectively). The cycle progresses clockwise from 0° (in-phase) to 180° (out-of-phase) to 360°/0°. Events correspond to bursts in lower record of **(D)** identified with vertical dotted bars. Timing is consistent with LLA. Arrow length represents the concentration (r) about the mean angle (Ø). The inner circle inside the phase diagram denotes the critical r vector calculated from the Rayleigh's Z table using α = 0.05 (Zar, 1999).

Eight dual experiments were conducted with pairs of spinal cords from littermates recorded simultaneously in the same bath chamber (*n* = 16 total sample). In the presence of NMDA and cycloheximide, motor rhythms with epochs consistent with a locomotor-like coordination were observed in all 16 spinal cords. Activity was comparatively fast at 0.31 ± 0.09 Hz but appeared to be weaker and more variable than that seen with the monoamines. Cycloheximide was applied before NMDA (*n* = 2), after NMDA (*n* = 5), or co-applied with NMDA (*n* = 1). Cycloheximide alone produced no observable action, but episodic LLA emerged with subsequent application of NMDA (Figure 12A). NMDA applied alone generated some epochs of weak bursting activity in at least one of the paired cords in 3/5 experiments with subsequent addition of cycloheximide leading to episodic LLA on spinal cord pairs (Figure 12B). In the 3 experiments with only bath applied NMDA, spontaneous bursting was also seen prior to NMDA application. Overall increases in initial cord excitability seen may have been due to residual cycloheximide from prior days. In one of these experiments, NMDA alone generated robust bursting though subsequent application of cycloheximide led to a clearly strengthened and regularized LLA (Figure 12C).

As described earlier, episodic tyramine- and PEA-evoked an episodic form of LLA never seen with the classical monoamines (in 33 and 70% of animals, respectively; Figure 7). After cycloheximide, episodic bouts of LLA appeared in 69% (11/16) of tested animals, 4 of which expressed rhythms that were distinctly PEA-like (Figure 12D). LLA coordination across recorded ventral roots is depicted in a polar plot of relative burst timing (Figure 12E). Thus, in the absence of protein synthesis, an increased bioavailable source of amino acids enabled the expression of episodic LLA, a distinctive feature of TA-evoked motor rhythms.

## Discussion

The neonatal rat spinal cord was shown to contain AADC, the essential synthesis enzyme for TA biosynthesis from their amino acid precursors, as well as their cognate G protein-coupled receptors (TAAR1 and TAAR4) that together define the essential substrates to exert biological actions. Whether and how this intrinsic spinal aminergic modulatory system is recruited is unknown, but exogenously provided TAs clearly promote motor pattern generating circuits. To exert their modulatory actions TAs show dependence on intracellular translocation via Na^+^-independent transporters, consistent with prominent intracellularly located TAARs. Unlike the classical monoamines, the TAs can also generate an episodic form of LLA. That episodic motor rhythms are also seen following increases in precursor amino acid availability supports the endogenous TA biosynthesis as a means to promote motor circuit activation.

### Anatomical observations

#### AADC and tyramine expression in the spinal cord

In both adult and neonate, we regularly observed AADC labeling in cells surrounding the central canal (D cells), in a ventral stream from this region, and in blood vessels. Central canal-related labeling is consistent with previous reports in rat, mouse, and monkey (Jaeger et al., 1983; Nagatsu et al., 1988; Barraud et al., 2010; Li et al., 2014; Wienecke et al., 2014). Labeling in blood vessels has also been reported (Jaeger et al., 1983; Li et al., 2014). AADC immunolabeling was also seen in other gray matter areas and in a midline ventral funiculus white matter bundle (Wienecke et al., 2014). This white matter tract likely represents axon projections of D cells as we observed that DiI applied to this region retrogradely labeled central canal cells. In the neonate, we also observed a ventral collection of AADC^+^ midline cells emanating from the central canal. These cells may represent a ventral “migratory stream” of active neurogenesis since Wienecke et al. (2014) observed that D cells are Neun^−^/doublecortin^+^ in adult rat, and thus likely to be newly generated neurons or neural precursors. Interestingly, motoneurons appeared to be weakly AADC^+^ though neither *in situ* hybridization nor immunodetection of AADC has been previously reported in rat spinal motoneurons. To support this observation, we mined our earlier microarray expression profiling study on laser capture microdissected medial and lateral motor column motoneurons (Cui et al., 2006). Using the present/absent call in Affymetrix Microarray Suite software, AADC cDNA was detected as present in all samples of motoneurons. In comparison, tyrosine hydroxylase and dopamine-β-hydroxylase cDNA were reported as absent.

Tyramine immunolabeling was also seen in AADC-containing neurons, consistent with AADC-mediated synthesis from tyrosine. It is assumed that tryptamine and PEA would also be synthesized by AADC from their respective amino acid precursors, but could not identify commercially available antibodies with acceptable specificity for these TAs. While the most consistent labeling observed for tyramine and AADC was in central canal midline regions and motoneurons, we noted considerable inter-animal variability. Indeed, the TAs have been described as protean. Metabolic sensitivity to temporal shifts in substrate availability may be a defining feature of the AADC-TA-TAAR modulatory system (Burchett and Hicks, 2006).

#### Trace amine-associated receptors 1 and 4 in the spinal cord

Previously, *in situ* hybridization, RT-PCR, and LacZ reporter expression studies all observed labeled TAAR1 in the brain (Borowsky et al., 2001; Lindemann et al., 2008) and one report also examined and detected expression in spinal cord (Borowsky et al., 2001). While TAARs are thought to be G_s_-coupled (Borowsky et al., 2001), there is also evidence of activation of G_q_-coupled signal transduction pathways (Panas et al., 2012). Here, RT-PCR identified expression of several TAARs in the neonatal rat spinal cord, including TAAR1 and TAAR4. We also observed TAAR1 and TAAR4 immunolabeling in neurons near the central canal and in motoneurons. TAAR1 and TAAR4 expression observed in the spinal cord overlapped with the expression of AADC and tyramine. Since tyramine and PEA activate TAAR1 and tryptamine and PEA activate TAAR4 (Borowsky et al., 2001), the TAs have a substrate for biological actions in the spinal cord. Suggestive immunolabeling evidence of a cytoplasmic location of both ligand (tyramine) and receptors (TAAR1 and TAAR4) supports an intracellular activation of signal transduction pathways (Miller, 2011). This is fully consistent with exogenously applied TAs showing dependence on transmembrane transport and taking longer than 5-HT and NA to exert modulatory actions. In summary, the mechanisms for TA synthesis and actions are anatomically coincident, and provide a substrate by which TAs can produce effects on their own.

### Electrophysiological actions

When applied alone, the monoamines, tryptamine, and tyramine increased motor activity, including via direct excitatory actions on motoneurons. This agrees with a previous study showing that tyramine can act directly on motoneurons (Kitazawa et al., 1985). PEA is an agonist at both TAAR1 and TAAR4 receptors (Borowsky et al., 2001; Bunzow et al., 2001); so their expression in motoneurons should also facilitate motor activity. However, no overt actions were observed. One possibility is that there is no membrane transporter for PEA in motoneurons. Another possibility is that TAARs are expressed as heterodimers (e.g., TAAR1/TAAR4) that preferentially interfere with PEA binding (Babusyte et al., 2013). Other possibilities include actions on other receptors (e.g., G_i_-coupled α_2_-adrenergic) that compete with TAAR1 and TAAR4 mediated actions (Pacifico et al., 2012).

We observed that 5-HT, NA, and DA produced comparable LLA in the presence of NMDA, consistent with reports of sub-locomotor doses of NMDA helping to stabilize and regularize the locomotor rhythm (Sqalli-Houssaini et al., 1993; Cowley and Schmidt, 1994; Kjaerulff et al., 1994; Schmidt et al., 1998). The TAs also produced LLA indistinguishable from that seen with the monoamine transmitters, strongly suggests that the TAs are acting at the level of the locomotor central pattern generator (CPG) and recruiting the same pattern-generating circuits. However, the detailed pattern of activation (which CPG neuron classes are recruited) may not be the same.

The TAs were also generated an episodic form of LLA not seen with the monoamine transmitters, and both continuous and episodic locomotor phenotypes could be observed at different times within an individual animal. Episodic LLA may represent a physiologic pattern recruited by endogenous mechanisms. For example, voluntary wheel running in rats and mice is episodic, occurring in short bouts separated by longer periods (Hanagasioglu and Borbely, 1982; Eikelboom and Mills, 1988; De Bono et al., 2006). Whelan et al. (2000) reported spontaneous episodes of rhythmic ventral root activity in the mouse very similar to those seen here (Whelan et al., 2000).

**Figure 13:**
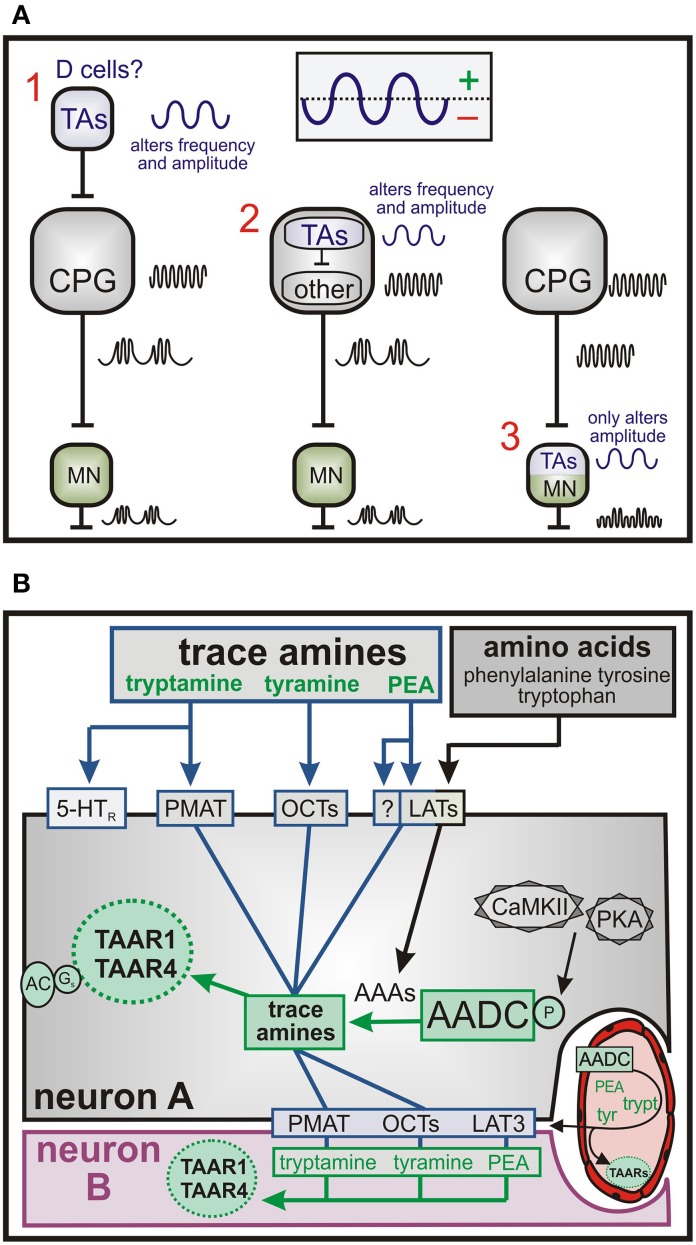
**Possible network and cellular pathways for TA-mediated actions. (A)** Possible location of TA modulatory actions in the emergence of episodic LLA. **(1)** Episodic bursts upstream of the locomotor central pattern generator (CPG) could include alternating excitation and inhibition. Putative trace aminergic neurons are the AADC^+^ D cells. Their location is within the spinal region responsible for locomotor rhythmogenesis. Their effect could be to generate a waxing and waning of CPG output to motoneurons (MN). **(2)** Modulatory actions within a subclass of CPG neurons. For example, the synaptic drive provided by the CPG could interact with slow frequency intrinsic voltage oscillations. **(3)** Modulatory actions within motoneurons are not consistent with observed waxing and waning in locomotor CPG frequency. **(B)** Putative transport and intracellular signaling mechanisms for TA actions. The TAs, tyramine and tryptamine, undergo Na^+^-independent membrane transport via the PCTs and PMAT, respectively. PEA transport could include the LATs, which are also responsible for transport of the precursor aromatic amino acids (AAAs), tyrosine, phenylalanine, and tryptophan. It is also possible for these TAs to be made from their AAA precursors in AADC-containing cells. Once inside, the TAs act on intracellularly-located TAARs to produce Gs-coupled neuromodulatory responses. The TAs could also be generated via AADC in some neurons (here neuron A) or from endothelia then transported into adjacent non-AADC but TAAR-containing neurons to exert neuromodulatory actions. Abbreviations: PMAT, plasma membrane monoamines transporter; OCTs, organic cat ion transporters; LATs, large neutral amino acid transporters; PKA, protein kinase A; cAMKII, calcium/calmodulin dependent protein kinase II; AC, adenylate cyclase; 5-HTR, 5-HT receptor; TA, trace amine; TAAR, trace amine-associated receptor.

Episodic LLA patterns may reflect recruitment of additional neurons or intrinsic membrane properties that influence the output of the spinal locomotor CPG. This is explored theoretically in Figure 13A with the assumption that the TA-induced regular LLA pattern is produced at the CPG level. Episodic modulation of LLA might occur in neurons that project onto the CPG (left illustration) themselves producing a much slower rhythmic synaptic drive onto the CPG. This is consistent with observed waxing and waning of LLA. Obvious candidate neurons are the AADC-expressing D cells surrounding the central canal (described below). Other possible organizations are depicted and explored in Figure 13A.

While the function of these D cells in the mammal is still unknown, similarly-located and projecting CSF-contacting neurons in larval zebrafish initiate slow swimming by optogenetic stimulation. Their genetic silencing reduced the frequency of spontaneous locomotion, and they provided the necessary tone for spontaneous forward swimming (Wyart et al., 2009). Based on the similarity in location, it is not unreasonable to consider a comparable role for D cells in the mammal. Aside from CSF-contacting D cells, many other AADC^+^ neurons were located in adjacent ventromedial locations. This location is consistent with ventromedially located interneurons shown to undergo intrinsic membrane voltage oscillations including in association with neurochemicals that induce LLA (Hochman et al., 1994; Tazerart et al., 2008; Brocard et al., 2013). Thus, D cells, with projections into the ventral funiculus, and previously reported synaptic projections toward motor nuclei (Jaeger et al., 1983) represent a likely source of the episodic modulation of locomotor activity.

### TA actions are mechanistically distinct from the monoamines transmitters

The TAs are structurally similar to the classical monoamines and may act as monoamine receptor agonists. Tryptamine can activate 5-HT_2_ and 5-HT_7_ receptors (Boess and Martin, 1994), on which 5-HT induced locomotion is dependent (Madriaga et al., 2004; Liu and Jordan, 2005; Liu et al., 2009), and tryptamine and 5-HT evoke similar locomotor patterns with similar sensitivity to methysergide block. On the other hand, tyramine and PEA-evoked LLA appear to have very low affinity to the monoamine receptors (U'Prichard et al., 1977; Shen et al., 1993; Peddi et al., 2003). Critically, the TAs including tryptamine require 50–100-fold longer incubations periods to activate LLA and this difference is not consistent actions via plasma membrane monoamine receptors. Therefore, tryptamine may require activation of 5HT as well as TAARs to generate LLA.

Slower actions were not due to indirect release of monoamines through their transporters since TA-induced LLA remained following block of VMAT and the Na^+^-dependent monoamine transporters. Instead, prolonged incubations periods are consistent with observed dependence of tyramine- and tryptamine-evoked LLA on intracellular transport via low affinity Na^+^-independent membrane transporters. Collectively these observations support TA recruitment of LLA by intrinsic spinal mechanisms independent of descending monoaminergic systems.

Notably, DA also took much longer to initiate LLA. Since DA can be generated in endothelia subsequent to hypoxic stress (Pfeil et al., 2014) and DA is a low affinity agonist at TAAR1 in rat (Bunzow et al., 2001), DA-induced LLA may normally associate with vascular release and subsequent intracellular neuronal transport via PMAT.

Dependence on transmembrane transport for PEA-evoked actions was not tested. Biologically, PEA transport is known to occur via a saturable proton-dependent transport process independent of all currently studied OCTs (Fischer et al., 2010). PEA is also a potent cis-transport inhibitor of PMAT (Ho et al., 2011). Some PEA transport may occur by Na^+^-independent system L amino acid transporter LAT3 (Babu et al., 2003), which shows widespread expression in the mouse spinal cord (Allen_Spinal_Cord_Atlas, 2009), as well as by simple diffusion (Berry et al., 2013).

As reported for TAAR1 in HEK cells (Bunzow et al., 2001; Miller, 2011), we observed cytoplasmic labeling for TAAR1 and TAAR4, both of which are activated by the TAs (Borowsky et al., 2001). A cytoplasmic location of the ligand and the receptor (e.g., tyramine and TAAR1) would support intracellular activation of signal transduction pathways (Miller, 2011). Such a co-localization would not require release from vesicles and could explain why the TAs do not appear to be found there (Berry, 2004; Burchett and Hicks, 2006).

Putative transport mechanisms for the TA are shown in Figure 13B. The TAs, tyramine and tryptamine, can transport via OCTs and PMAT, respectively, whereas PEA may transport via LAT3 but more likely via a currently unidentified transporter. TA biosynthesis can alternatively be generated intracellularly from aromatic amino acid (AAA) precursors in AADC-containing cells to act on intracellularly-located TAARs. This would constitute a form of biochemical integration (Katz and Clemens, 2001). TAs generated via AADC in some neurons (here neuron A) or from endothelia can transport into adjacent non-AADC but TAAR-containing neurons to exert neuromodulatory actions (OCTs and PMAT transport is bidirectional). Last, as AADC and TAARs are present in vasculature, TA modulation of vascular function may lead to secondary actions on neurons (Hardebo et al., 1979; Nagatsu et al., 1988; Broadley, 2010; Anwar et al., 2012).

### TA biosynthesis as a sympathetic autonomic cellular stress response

Intrinsic spinal cord AADC activity should enable endogenous synthesis of the TAs from their AAA precursors. However, when the AAAs were bath-applied, no obvious maintained motor rhythms were observed. Since intracellular transport of AAAs may be rapidly sequestered for protein synthesis during this neonatal period of dramatic growth, we blocked protein synthesis with cycloheximide in order to increase biochemically access to AAAs (Beugnet et al., 2003). The effects of cycloheximide were dramatic with LLA developing in all animals. Additional studies are required to directly link increases in AAAs to increases in endogenous TA biosynthesis to induce a neuromodulatory response. However, emergent LLA included TA-like episodic bouts including those with burst structures notably comparable to that seen with PEA.

Access to precursor amino acids for TA biosynthesis may depend heavily stress-induced protein catabolism. As the TAs are known sympathomimetics (Branchek and Blackburn, 2003; Berry, 2004), spinal TAs may function to facilitate motor responses during strong sympathetic nervous system activation. For example, marked PEA increases are seen in human urine after a highly stressful event (Paulos and Tessel, 1982). Just as DA can be generated via AADC in endothelia subsequent to hypoxic stress (Pfeil et al., 2014), intracellularly-synthesized TAs may comprise an integral physiological component of the autonomic stress response.

### Conflict of interest statement

The authors declare that the research was conducted in the absence of any commercial or financial relationships that could be construed as a potential conflict of interest.

## References

[B1] Abbas A. K. (2013). Evidence for constitutive protein synthesis in hippocampal LTP stabilization. Neuroscience 246, 301–311. 10.1016/j.neuroscience.2013.05.011 23685165

[B2] Allen_Spinal_Cord_Atlas. (2009). Allen Institute for Brain Science [Online]. Seattle, WA. Available online at: http://mousespinal.brain-map.org

[B2a] Ambalavanar R. Morris R. (1989). Fluoro-Gold injected either subcutaneously or intravascularly results in extensive retrograde labelling of CNS neurones having axons terminating outside the blood-brain barrier. Brain res. 505, 171–175. 10.1016/0006-8993(89)90133-9 2611674

[B3] Anwar M. A. Ford W. R. Broadley K. J. Herbert A. A. (2012). Vasoconstrictor and vasodilator responses to tryptamine of rat-isolated perfused mesentery: comparison with tyramine and beta-phenylethylamine. Br. J. Pharmacol. 165, 2191–2202. 10.1111/j.1476-5381.2011.01706.x 21958009 PMC3413856

[B4] Babu E. Kanai Y. Chairoungdua A. Kim D. K. Iribe Y. Tangtrongsup S.. (2003). Identification of a novel system L amino acid transporter structurally distinct from heterodimeric amino acid transporters. J. Biol. Chem. 278, 43838–43845. 10.1074/jbc.M305221200 12930836

[B5] Babusyte A. Kotthoff M. Fiedler J. Krautwurst D. (2013). Biogenic amines activate blood leukocytes via trace amine-associated receptors TAAR1 and TAAR2. J. Leukoc. Biol. 93, 387–394. 10.1189/jlb.0912433 23315425

[B6] Barraud Q. Obeid I. Aubert I. Barriere G. Contamin H. McGuire S.. (2010). Neuroanatomical study of the A11 diencephalospinal pathway in the non-human primate. PLoS ONE 5:e13306. 10.1371/journal.pone.0013306 20967255 PMC2954154

[B7] Barriere G. Mellen N. Cazalets J. R. (2004). Neuromodulation of the locomotor network by dopamine in the isolated spinal cord of newborn rat. Eur. J. Neurosci. 19, 1325–1335. 10.1111/j.1460-9568.2004.03210.x 15016090

[B8] Berry M. D. (2004). Mammalian central nervous system trace amines. Pharmacologic amphetamines, physiologic neuromodulators. J. Neurochem. 90, 257–271. 10.1111/j.1471-4159.2004.02501.x 15228583

[B9] Berry M. D. (2007). The potential of trace amines and their receptors for treating neurological and psychiatric diseases. Rev. Recent Clin. Trials 2, 3–19. 10.2174/157488707779318107 18473983

[B10] Berry M. D. Shitut M. R. Almousa A. Alcorn J. Tomberli B. (2013). Membrane permeability of trace amines: evidence for a regulated, activity-dependent, nonexocytotic, synaptic release. Synapse 67, 656–667. 10.1002/syn.21670 23564683

[B11] Beugnet A. Tee A. R. Taylor P. M. Proud C. G. (2003). Regulation of targets of mTOR (mammalian target of rapamycin) signalling by intracellular amino acid availability. Biochem. J. 372, 555–566. 10.1042/BJ20021266 12611592 PMC1223408

[B12] Boess F. G. Martin I. L. (1994). Molecular biology of 5-HT receptors. Neuropharmacology 33, 275–317. 10.1016/0028-3908(94)90059-0 7984267

[B13] Borowsky B. Adham N. Jones K. A. Raddatz R. Artymyshyn R. Ogozalek K. L.. (2001). Trace amines: identification of a family of mammalian G protein-coupled receptors. Proc. Natl. Acad. Sci. U.S.A. 98, 8966–8971. 10.1073/pnas.151105198 11459929 PMC55357

[B14] Boulton A. A. (1976). Identification, distribution, metabolism, and function of meta and para tyramine, phenylethylamine and tryptamine in brain. Adv. Biochem. Psychopharmacol. 15, 57–67. 799463

[B15] Boulton A. A. (1978). The tyramines: functionally significant biogenic amines or metabolic accidents? Life Sci. 23, 659–671. 10.1016/0024-3205(78)90064-4 357877

[B16] Boulton A. A. (1991). Phenylethylaminergic modulation of catecholaminergic neurotransmission. Prog. Neuropsychopharmacol. Biol. Psychiatry 15, 139–156. 10.1016/0278-5846(91)90076-D 1651528

[B17] Boulton A. A. Juorio A. V. Paterson I. A. (1990). Phenylethylamine in the CNS: effects of monoamine oxidase inhibiting drugs, deuterium substitution and lesions and its role in the neuromodulation of catecholaminergic neurotransmission. J. Neural Transm. Suppl. 29, 119–129. 2193105 10.1007/978-3-7091-9050-0_12

[B18] Boulton A. A. Juorio A. V. Philips S. R. Wu P. H. (1977). The effects of reserpine and 6-hydroxydopamine on the concentrations of some arylakylamines in rat brain. Br. J. Pharmacol. 59, 209–214. 10.1111/j.1476-5381.1977.tb06996.x 837000 PMC1667690

[B19] Bowman W. C. Callingham B. A. Osuide G. (1964). EFFECTS OF TYRAMINE ON A SPINAL REFLEX IN THE ANAESTHETISED CHICK. J. Pharm. Pharmacol. 16, 505–515. 10.1111/j.2042-7158.1964.tb07505.x 14221186

[B20] Bradaia A. Trube G. Stalder H. Norcross R. D. Ozmen L. Wettstein J. G.. (2009). The selective antagonist EPPTB reveals TAAR1-mediated regulatory mechanisms in dopaminergic neurons of the mesolimbic system. Proc. Natl. Acad. Sci. U.S.A. 106, 20081–20086. 10.1073/pnas.0906522106 19892733 PMC2785295

[B21] Branchek T. A. Blackburn T. P. (2003). Trace amine receptors as targets for novel therapeutics: legend, myth and fact. Curr. Opin. Pharmacol. 3, 90–97. 10.1016/S1471-4892(02)00028-0 12550748

[B22] Broadley K. J. (2010). The vascular effects of trace amines and amphetamines. Pharmacol. Ther. 125, 363–375. 10.1016/j.pharmthera.2009.11.005 19948186

[B23] Brocard F. Shevtsova N. A. Bouhadfane M. Tazerart S. Heinemann U. Rybak I. A.. (2013). Activity-dependent changes in extracellular Ca^2+^ and K^+^ reveal pacemakers in the spinal locomotor-related network. Neuron 77, 1047–1054. 10.1016/j.neuron.2013.01.026 23522041 PMC3736142

[B24] Bunzow J. R. Sonders M. S. Arttamangkul S. Harrison L. M. Zhang G. Quigley D. I.. (2001). Amphetamine, 3,4-methylenedioxy methamphetamine, lysergic acid diethylamide, and metabolites of the catecholamine neurotransmitters are agonists of a rat trace amine receptor. Mol. Pharmacol. 60, 1181–1188. 10.1124/mol.60.6.1181 11723224

[B25] Burchett S. A. Hicks T. P. (2006). The mysterious trace amines: protean neuromodulators of synaptic transmission in mammalian brain. Prog. Neurobiol. 79, 223–246. 10.1016/j.pneurobio.2006.07.003 16962229

[B26] Cazalets J. R. Borde M. Clarac F. (1995). Localization and organization of the central pattern generator for hindlimb locomotion in newborn rat. J. Neurosci. 15, 4943–4951. 7623124 10.1523/JNEUROSCI.15-07-04943.1995 PMC6577873

[B27] Chiellini G. Frascarelli S. Ghelardoni S. Carnicelli V. Tobias S. C. Debarber A.. (2007). Cardiac effects of 3-iodothyronamine: a new aminergic system modulating cardiac function. FASEB J. 21, 1597–1608. 10.1096/fj.06-7474com 17284482

[B28] Clarac F. Pearlstein E. Pflieger J. F. Vinay L. (2004). The *in vitro* neonatal rat spinal cord preparation: a new insight into mammalian locomotor mechanisms. J. Comp. Physiol. A Neuroethol. Sens. Neural Behav. Physiol. 190, 343–357. 10.1007/s00359-004-0499-2 14872261

[B29] Cowley K. C. Schmidt B. J. (1994). Some limitations of ventral root recordings for monitoring locomotion in the *in vitro* neonatal rat spinal cord preparation. Neurosci. Lett. 171, 142–146. 10.1016/0304-3940(94)90625-4 8084476

[B30] Cowley K. C. Schmidt B. J. (1997). Regional distribution of the locomotor pattern-generating network in the neonatal rat spinal cord. J. Neurophysiol. 77, 247–259. 9120567 10.1152/jn.1997.77.1.247

[B30a] Cui D. Dougherty K. J. Machacek D. W. Sawchuk M. Hochman S. Baro D. J. (2006). Divergence between motoneurons: gene expression profiling provides a molecular characterization of functionally discrete somatic and autonomic motoneurons. Physiol. Genomics 24, 276–289. 10.1152/physiolgenomics.00109.2005 16317082 PMC2724224

[B31] De Bono J. P. Adlam D. Paterson D. J. Channon K. M. (2006). Novel quantitative phenotypes of exercise training in mouse models. Am. J. Physiol. Regul. Integr. Comp. Physiol. 290, R926–R934. 10.1152/ajpregu.00694.2005 16339385

[B32] Durden D. A. Philips S. R. Boulton A. A. (1973). Identification and distribution of beta-phenylethylamine in the rat. Can. J. Biochem. 51, 995–1002. 10.1139/o73-129 4725364

[B33] Eikelboom R. Mills R. (1988). A microanalysis of wheel running in male and female rats. Physiol. Behav. 43, 625–630. 10.1016/0031-9384(88)90217-X 3200918

[B34] Engel K. Wang J. (2005). Interaction of organic cations with a newly identified plasma membrane monoamine transporter. Mol. Pharmacol. 68, 1397–1407. 10.1124/mol.105.016832 16099839

[B35] Fischer W. Neubert R. H. Brandsch M. (2010). Transport of phenylethylamine at intestinal epithelial (Caco-2) cells: mechanism and substrate specificity. Eur. J. Pharm. Biopharm. 74, 281–289. 10.1016/j.ejpb.2009.11.014 19962438

[B36] Garcia-Ramirez D. L. Calvo J. R. Hochman S. Quevedo J. N. (2014). Serotonin, dopamine and noradrenaline adjust actions of myelinated afferents via modulation of presynaptic inhibition in the mouse spinal cord. PLoS ONE 9:e89999. 10.1371/journal.pone.0089999 24587177 PMC3938568

[B37] Gerin C. Becquet D. Privat A. (1995). Direct evidence for the link between monoaminergic descending pathways and motor activity. 1. A study with microdialysis probes implanted in the ventral funiculus of the spinal cord. Brain Res. 704, 191–201. 10.1016/0006-8993(95)01111-0 8788914

[B38] Gieseker E. Williams K. Sawchuk M. Hochman S. (2004). The trace amine tyramine is found in spinal ventral horn neurons and induces locomotor-like activity in the isolated neonatal rat spinal cord. Soc. Neurosci. Abstr. 30.

[B39] Gozal E. A. Sawchuk M. A. Hochman S. (2007). Trace amine immunolabeling and motor patterning in the neonatal rat spinal cord. Soc. Neurosci. Abstr. 33.

[B40] Gozal E. A. Williams K. Sawchuk M. A. Hochman S. (2006). Trace amines recruit motor activity in the isolated neonatal rat spinal cord. Soc. Neurosci. Abstr. 32. 10444672

[B41] Hanagasioglu M. Borbely A. A. (1982). Effect of voluntary locomotor activity on sleep in the rat. Behav. Brain Res. 4, 359–368. 10.1016/0166-4328(82)90060-2 7073885

[B42] Hardebo J. E. Falck B. Owman C. Rosengren E. (1979). Studies on the enzymatic blood-brain barrier: quantitative measurements of DOPA decarboxylase in the wall of microvessels as related to the parenchyma in various CNS regions. Acta Physiol. Scand. 105, 453–460. 10.1111/j.1748-1716.1979.tb00110.x 452922

[B43] Hayes H. B. Chang Y. H. Hochman S. (2009). An *in vitro* spinal cord-hindlimb preparation for studying behaviorally relevant rat locomotor function. J. Neurophysiol. 101, 1114–1122. 10.1152/jn.90523.2008 19073815 PMC2657055

[B44] Ho H. T. Pan Y. Cui Z. Duan H. Swaan P. W. Wang J. (2011). Molecular analysis and structure-activity relationship modeling of the substrate/inhibitor interaction site of plasma membrane monoamine transporter. J. Pharmacol. Exp. Ther. 339, 376–385. 10.1124/jpet.111.184036 21816955 PMC3199987

[B45] Hochman S. Garraway S. M. Machacek D. W. Shay B. L. (2001). 5-HT receptors and the neuromodulatory control of spinal cord function, in Motor Neurobiology of the Spinal Cord, ed Cope T. C. (Boca Raton: CRC Press), 47–87.

[B46] Hochman S. Gozal E. A. Hayes H. B. Anderson J. T. Deweerth S. P. Chang Y. H. (2012). Enabling techniques for *in vitro* studies on mammalian spinal locomotor mechanisms. Front. Biosci. 17, 2158–2180. 10.2741/4043 22652770 PMC7001871

[B47] Hochman S. Jordan L. M. Macdonald J. F. (1994). N-methyl-D-aspartate receptor-mediated voltage oscillations in neurons surrounding the central canal in slices of rat spinal cord. J. Neurophysiol. 72, 565–577. 7983519 10.1152/jn.1994.72.2.565

[B48] Jacobs B. L. Fornal C. A. (1993). 5-HT and motor control: a hypothesis. Trends Neurosci. 16, 346–352. 10.1016/0166-2236(93)90090-9 7694403

[B49] Jaeger C. B. Ruggiero D. A. Albert V. R. Joh T. H. Reis D. J. (1984). Immunocytochemical localization of aromatic-L-amino acid decarboxylase, in Handbook of Chemical Neuroanatomy, Vol. 2, Classical Transmitters in the CNS, Part 1, eds Bjorkland A. Hokfelt T. (Amsterdam: Elsevier), 387–408.

[B50] Jaeger C. B. Teitelman G. Joh T. H. Albert V. R. Park D. H. Reis D. J. (1983). Some neurons of the rat central nervous system contain aromatic-L-amino-acid decarboxylase but not monoamines. Science 219, 1233–1235. 10.1126/science.6131537 6131537

[B51] Jonker J. W. Schinkel A. H. (2004). Pharmacological and physiological functions of the polyspecific organic cation transporters: OCT1, 2, and 3 (SLC22A1-3). J. Pharmacol. Exp. Ther. 308, 2–9. 10.1124/jpet.103.053298 14576340

[B52] Jung N. Lehmann C. Rubbert A. Knispel M. Hartmann P. Van Lunzen J.. (2008). Relevance of the organic cation transporters 1 and 2 for antiretroviral drug therapy in human immunodeficiency virus infection. Drug Metab. Dispos. 36, 1616–1623. 10.1124/dmd.108.020826 18490433

[B53] Juorio A. V. (1979). Drug-induced changes in the formation, storage and metabolism of tyramine in the mouse. Br. J. Pharmacol. 66, 377–384. 10.1111/j.1476-5381.1979.tb10841.x 43172 PMC2043695

[B54] Juorio A. V. (1980). Effects of molindone and fluphenazine on the brain concentration of some phenolic and catecholic amines in the mouse and the rat. Br. J. Pharmacol. 70, 475–480. 10.1111/j.1476-5381.1980.tb08726.x 6777007 PMC2044360

[B55] Juorio A. V. (1988). Brain beta-phenylethylamine: localization, pathways, and interrelation with catecholamines, in Progress in Catecholamine Research, eds Dahlstrom A. Sandler M. Belmarker R. (New York, NY: Alan R. Liss), 433–437.

[B56] Karoum F. Nasrallah H. Potkin S. Chuang L. Moyer-Schwing J. Phillips I.. (1979). Mass fragmentography of phenylethylamine, m- and p-tyramine and related amines in plasma, cerebrospinal fluid, urine, and brain. J. Neurochem. 33, 201–212. 10.1111/j.1471-4159.1979.tb11722.x 458449

[B57] Katz P. S. Clemens S. (2001). Biochemical networks in nervous systems: expanding neuronal information capacity beyond voltage signals. Trends Neurosci. 24, 18–25. 10.1016/S0166-2236(00)01686-6 11163883

[B58] Kiehn O. (2006). Locomotor circuits in the mammalian spinal cord. Annu. Rev. Neurosci. 29, 279–306. 10.1146/annurev.neuro.29.051605.112910 16776587

[B59] Kiehn O. Kjaerulff O. (1996). Spatiotemporal characteristics of 5-HT and dopamine-induced rhythmic hindlimb activity in the *in vitro* neonatal rat. J. Neurophysiol. 75, 1472–1482. 8727391 10.1152/jn.1996.75.4.1472

[B60] Kiehn O. Kjaerulff O. (1998). Distribution of central pattern generators for rhythmic motor outputs in the spinal cord of limbed vertebrates. Ann. N.Y. Acad. Sci. 860, 110–129. 10.1111/j.1749-6632.1998.tb09043.x 9928306

[B61] Kiehn O. Sillar K. T. Kjaerulff O. McDearmid J. R. (1999). Effects of noradrenaline on locomotor rhythm-generating networks in the isolated neonatal rat spinal cord. J. Neurophysiol. 82, 741–746. 10444672 10.1152/jn.1999.82.2.741

[B62] Kitazawa T. Saito K. Ohga A. (1985). Effects of catecholamines on spinal motoneurones and spinal reflex discharges in the isolated spinal cord of the newborn rat. Brain Res. 351, 31–36. 10.1016/0165-3806(85)90228-7 3995339

[B63] Kjaerulff O. Barajon I. Kiehn O. (1994). Sulphorhodamine-labelled cells in the neonatal rat spinal cord following chemically induced locomotor activity *in vitro*. J. Physiol. 478(Pt 2), 265–273. 7525942 10.1113/jphysiol.1994.sp020248 PMC1155684

[B64] Kjaerulff O. Kiehn O. (1996). Distribution of networks generating and coordinating locomotor activity in the neonatal rat spinal cord *in vitro*: a lesion study. J. Neurosci. 16, 5777–5794. 8795632 10.1523/JNEUROSCI.16-18-05777.1996 PMC6578971

[B65] Kremer E. Lev T. A. (1997). Localization of the spinal network associated with generation of hindlimb locomotion in the neonatal rat and organization of its transverse coupling system. J. Neurophysiol. 77, 1155–1170. 9084588 10.1152/jn.1997.77.3.1155

[B66] Leo D. Mus L. Espinoza S. Hoener M. C. Sotnikova T. D. Gainetdinov R. R. (2014). Taar1-mediated modulation of presynaptic dopaminergic neurotransmission: role of D2 dopamine autoreceptors. Neuropharmacology 81, 283–291. 10.1016/j.neuropharm.2014.02.007 24565640

[B67] Li Y. Li L. Stephens M. J. Zenner D. Murray K. C. Winship I. R.. (2014). Synthesis, transport, and metabolism of serotonin formed from exogenously applied 5-HTP after spinal cord injury in rats. J. Neurophysiol. 111, 145–163. 10.1152/jn.00508.2013 24068759 PMC3921369

[B68] Lindemann L. Meyer C. A. Jeanneau K. Bradaia A. Ozmen L. Bluethmann H.. (2008). Trace amine-associated receptor 1 modulates dopaminergic activity. J. Pharmacol. Exp. Ther. 324, 948–956. 10.1124/jpet.107.132647 18083911

[B69] Liu J. Akay T. Hedlund P. B. Pearson K. G. Jordan L. M. (2009). Spinal 5-HT7 receptors are critical for alternating activity during locomotion: *in vitro* neonatal and *in vivo* adult studies using 5-HT7 receptor knockout mice. J. Neurophysiol. 102, 337–348. 10.1152/jn.91239.2008 19458153

[B70] Liu J. Jordan L. M. (2005). Stimulation of the parapyramidal region of the neonatal rat brain stem produces locomotor-like activity involving spinal 5-HT7 and 5-HT2A receptors. J. Neurophysiol. 94, 1392–1404. 10.1152/jn.00136.2005 15872068

[B71] Madriaga M. A. McPhee L. C. Chersa T. Christie K. J. Whelan P. J. (2004). Modulation of locomotor activity by multiple 5-HT and dopaminergic receptor subtypes in the neonatal mouse spinal cord. J. Neurophysiol. 92, 1566–1576. 10.1152/jn.01181.2003 15163678

[B72] Mandela P. Chandley M. Xu Y. Y. Zhu M. Y. Ordway G. A. (2010). Reserpine-induced reduction in norepinephrine transporter function requires catecholamine storage vesicles. Neurochem. Int. 56, 760–767. 10.1016/j.neuint.2010.02.011 20176067 PMC2859979

[B73] Mann R. Bell C. (1991). Neuronal metabolism and DOPA decarboxylase immunoreactivity in terminal noradrenergic sympathetic axons of rat. J. Histochem. Cytochem. 39, 663–668. 10.1177/39.5.1673138 1673138

[B74] Martin L. L. Baker G. B. Wood P. L. (1988). Tryptamine turnover: effects of drugs, in Trace Amines: Comparative and Clinical Neurobiology, eds Boulton A. A. Juorio A. V. Downer R. G. H. (New Jersey, NJ: Humana Press), 95–104, 57–174.

[B75] Matsushima T. Grillner S. (1992). Neural mechanisms of intersegmental coordination in lamprey: local excitability changes modify the phase coupling along the spinal cord. J. Neurophysiol. 67, 373–388. 1569465 10.1152/jn.1992.67.2.373

[B75a] Merchenthaler I. (1991). Neurons with access to the general circulation in the central nervous system of the rat: a retrograde tracing study with fluoro-gold. Neuroscience 44, 655–662. 10.1016/0306-4522(91)90085-3 1721686

[B76] Millan M. J. (2002). Descending control of pain. Prog. Neurobiol. 66, 355–474. 10.1016/S0301-0082(02)00009-6 12034378

[B77] Miller G. M. (2011). The emerging role of trace amine-associated receptor 1 in the functional regulation of monoamine transporters and dopaminergic activity. J. Neurochem. 116, 164–176. 10.1111/j.1471-4159.2010.07109.x 21073468 PMC3005101

[B78] Murray K. C. Stephens M. J. Ballou E. W. Heckman C. J. Bennett D. J. (2011). Motoneuron excitability and muscle spasms are regulated by 5-HT2B and 5-HT2C receptor activity. J. Neurophysiol. 105, 731–748. 10.1152/jn.00774.2010 20980537 PMC3059173

[B79] Nagatsu I. Sakai M. Yoshida M. Nagatsu T. (1988). Aromatic L-amino acid decarboxylase-immunoreactive neurons in and around the cerebrospinal fluid-contacting neurons of the central canal do not contain dopamine or serotonin in the mouse and rat spinal cord. Brain Res. 475, 91–102. 10.1016/0006-8993(88)90202-8 3214730

[B80] Nguyen T. V. Juorio A. V. (1989). Binding sites for brain trace amines. Cell. Mol. Neurobiol. 9, 297–311. 10.1007/BF00711411 2558802 PMC11567294

[B81] Ono H. Ito H. Fukuda H. (1991). 2-phenylethylamine and methamphetamine enhance the spinal monosynaptic reflex by releasing noradrenaline from the terminals of descending fibers. Jpn. J. Pharmacol. 55, 359–366. 10.1254/jjp.55.359 1830350

[B82] Pacifico R. Dewan A. Cawley D. Guo C. Bozza T. (2012). An olfactory subsystem that mediates high-sensitivity detection of volatile amines. Cell Rep. 2, 76–88. 10.1016/j.celrep.2012.06.006 22840399 PMC3408605

[B83] Panas M. W. Xie Z. Panas H. N. Hoener M. C. Vallender E. J. Miller G. M. (2012). Trace amine associated receptor 1 signaling in activated lymphocytes. J. Neuroimmune Pharmacol. 7, 866–876. 10.1007/s11481-011-9321-4 22038157 PMC3593117

[B84] Paulos M. A. Tessel R. E. (1982). Excretion of beta-phenethylamine is elevated in humans after profound stress. Science 215, 1127–1129. 10.1126/science.7063846 7063846

[B85] Peddi S. Roth B. L. Glennon R. A. Westkaemper R. B. (2003). Spiro[9,10-dihydroanthracene]-9,3′-pyrrolidine-a structurally unique tetracyclic 5-HT2A receptor antagonist. Eur. J. Pharmacol. 482, 335–337. 10.1016/j.ejphar.2003.09.059 14660041

[B86] Pfeil U. Kuncova J. Bruggmann D. Paddenberg R. Rafiq A. Henrich M.. (2014). Intrinsic vascular dopamine—a key modulator of hypoxia-induced vasodilation in splanchnic vessels. J. Physiol. 592(Pt 8), 1745–1756. 10.1113/jphysiol.2013.262626 24535440 PMC4001749

[B87] Philips S. R. Davis B. A. Durden D. A. Boulton A. A. (1975). Identification and distribution of m-tyramine in the rat. Can. J. Biochem. 53, 65–69. 10.1139/o75-010 1120292

[B88] Philips S. R. Durden D. A. Boulton A. A. (1974a). Identification and distribution of p-tyramine in the rat. Can. J. Biochem. 52, 366–373. 10.1139/o74-055 4838667

[B89] Philips S. R. Durden D. A. Boulton A. A. (1974b). Identification and distribution of tryptamine in the rat. Can. J. Biochem. 52, 447–451. 10.1139/o74-068 4844280

[B90] Premont R. T. Gainetdinov R. R. Caron M. G. (2001). Following the trace of elusive amines. Proc. Natl. Acad. Sci. U.S.A. 98, 9474–9475. 10.1073/pnas.181356198 11504935 PMC55475

[B91] Reddy S. V. Maderdrut J. L. Yaksh T. L. (1980). Spinal cord pharmacology of adrenergic agonist-mediated antinociception. J. Pharmacol. Exp. Ther. 213, 525–533. 6110767

[B92] Rekling J. C. Funk G. D. Bayliss D. A. Dong X. W. Feldman J. L. (2000). Synaptic control of motoneuronal excitability. Physiol. Rev. 80, 767–852. 10747207 10.1152/physrev.2000.80.2.767 PMC4764886

[B93] Revel F. G. Moreau J. L. Gainetdinov R. R. Bradaia A. Sotnikova T. D. Mory R.. (2011). TAAR1 activation modulates monoaminergic neurotransmission, preventing hyperdopaminergic and hypoglutamatergic activity. Proc. Natl. Acad. Sci. U.S.A. 108, 8485–8490. 10.1073/pnas.1103029108 21525407 PMC3101002

[B94] Reynolds I. J. Aizenman E. (1992). Pentamidine is an N -methyl-D-aspartate receptor antagonist and is neuroprotective *in vitro*. J. Neurosci. 12, 970–975. 1532027 10.1523/JNEUROSCI.12-03-00970.1992 PMC6576030

[B95] Reynolds I. J. Zeleski D. M. Rothermund K. D. Hartnett K. A. Tidwell R. Aizenman E. (1993). Studies on the effects of several pentamidine analogues on the NMDA receptor. Eur. J. Pharmacol. Mol. Pharmacol. 244, 175–179. 10.1016/0922-4106(93)90023-3 8432312

[B96] Saavedra J. M. (1989). á-phenylethylamine, phenylethanoamine, tyramine and octopamine, in Catecholamines II, eds Trendelenburg U. Weiner N. (Berlin: Springer-Verlag), 181–210.

[B97] Schmidt B. J. Hochman S. Maclean J. N. (1998). NMDA receptor-mediated oscillatory properties: potential role in rhythm generation in the mammalian spinal cord. Ann. N.Y. Acad. Sci. 860, 189–202. 10.1111/j.1749-6632.1998.tb09049.x 9928312

[B98] Schmidt B. J. Jordan L. M. (2000). The role of serotonin in reflex modulation and locomotor rhythm production in the mammalian spinal cord. Brain Res. Bull. 53, 689–710. 10.1016/S0361-9230(00)00402-0 11165804

[B99] Schomig E. Lazar A. Grundemann D. (2006). Extraneuronal monoamine transporter and organic cation transporters 1 and 2: a review of transport efficiency. Handb. Exp. Pharmacol. 151–180. 10.1007/3-540-29784-7_8 16722235

[B100] Shen Y. Monsma F. J. Jr. Metcalf M. A. Jose P. A. Hamblin M. W. Sibley D. R. (1993). Molecular cloning and expression of a 5-hydroxytryptamine7 serotonin receptor subtype. J. Biol. Chem. 268, 18200–18204. 8394362

[B101] Shreckengost J. Calvo J. Quevedo J. Hochman S. (2010). Bicuculline-sensitive primary afferent depolarization remains after greatly restricting synaptic transmission in the mammalian spinal cord. J. Neurosci. 30, 5283–5288. 10.1523/JNEUROSCI.3873-09.2010 20392950 PMC6632755

[B102] Spector S. Melmon K. Lovenberg W. Sjoerdsma A. (1963). The presence and distribution of tyramine in mammalian tissues. J. Pharmacol. Exp. Ther. 140, 229–235. 13989973

[B103] Sqalli-Houssaini Y. Cazalets J. R. (2000). Noradrenergic control of locomotor networks in the *in vitro* spinal cord of the neonatal rat. Brain Res. 852, 100–109. 10.1016/S0006-8993(99)02219-2 10661501

[B104] Sqalli-Houssaini Y. Cazalets J. R. Clarac F. (1993). Oscillatory properties of the central pattern generator for locomotion in neonatal rats. J. Neurophysiol. 70, 803–813. 8410173 10.1152/jn.1993.70.2.803

[B105] Tazerart S. Vinay L. Brocard F. (2008). The persistent sodium current generates pacemaker activities in the central pattern generator for locomotion and regulates the locomotor rhythm. J. Neurosci. 28, 8577–8589. 10.1523/JNEUROSCI.1437-08.2008 18716217 PMC6671046

[B106] Toll L. Berzetei-Gurske I. P. Polgar W. E. Brandt S. R. Adapa I. D. Rodriguez L.. (1998). Standard binding and functional assays related to medications development division testing for potential cocaine and opiate narcotic treatment medications. NIDA Res. Monogr. 178, 440–466. 9686407

[B107] Torres G. E. Gainetdinov R. R. Caron M. G. (2003). Plasma membrane monoamine transporters: structure, regulation and function. Nat. Rev. Neurosci. 4, 13–25. 10.1038/nrn1008 12511858

[B108] U'Prichard D. C. Greenberg D. A. Snyder S. H. (1977). Binding characteristics of a radiolabeled agonist and antagonist at central nervous system alpha noradrenergic receptors. Mol. Pharmacol. 13, 454–473. 17827

[B109] Whelan P. Bonnot A. O'Donovan M. J. (2000). Properties of rhythmic activity generated by the isolated spinal cord of the neonatal mouse. J. Neurophysiol. 84, 2821–2833. 11110812 10.1152/jn.2000.84.6.2821

[B110] Wienecke J. Ren L. Q. Hultborn H. Chen M. Moller M. Zhang Y.. (2014). Spinal cord injury enables aromatic l-amino acid decarboxylase cells to synthesize monoamines. J. Neurosci. 34, 11984–12000. 10.1523/JNEUROSCI.3838-13.2014 25186745 PMC6608456

[B111] Wolinsky T. D. Swanson C. J. Smith K. E. Zhong H. Borowsky B. Seeman P.. (2007). The Trace Amine 1 receptor knockout mouse: an animal model with relevance to schizophrenia. Genes Brain Behav. 6, 628–639. 10.1111/j.1601-183X.2006.00292.x 17212650

[B112] Wyart C. Del Bene F. Warp E. Scott E. K. Trauner D. Baier H.. (2009). Optogenetic dissection of a behavioural module in the vertebrate spinal cord. Nature 461, 407–410. 10.1038/nature08323 19759620 PMC2770190

[B113] Xie Z. Miller G. M. (2008). Beta-phenylethylamine alters monoamine transporter function via trace amine-associated receptor 1: implication for modulatory roles of trace amines in brain. J. Pharmacol. Exp. Ther. 325, 617–628. 10.1124/jpet.107.134247 18182557

[B114] Xie Z. Vallender E. J. Yu N. Kirstein S. L. Yang H. Bahn M. E.. (2008). Cloning, expression, and functional analysis of rhesus monkey trace amine-associated receptor 6: evidence for lack of monoaminergic association. J. Neurosci. Res. 86, 3435–3446. 10.1002/jnr.21783 18627029 PMC2644554

[B115] Xie Z. Westmoreland S. V. Bahn M. E. Chen G. L. Yang H. Vallender E. J.. (2007). Rhesus monkey trace amine-associated receptor 1 signaling: enhancement by monoamine transporters and attenuation by the D2 autoreceptor *in vitro*. J. Pharmacol. Exp. Ther. 321, 116–127. 10.1124/jpet.106.116863 17234900

[B116] Yamamoto H. Kamegaya E. Hagino Y. Imai K. Fujikawa A. Tamura K.. (2007). Genetic deletion of vesicular monoamine transporter-2 (VMAT2) reduces dopamine transporter activity in mesencephalic neurons in primary culture. Neurochem. Int. 51, 237–244. 10.1016/j.neuint.2007.06.022 17664021

[B117] Zar J. H. (1999). Biostatistical Analysis: Upper Saddle River. New Jersey, NJ: Prentice Hall.

[B117a] Zhu H. Clemens S. Sawchuk M. Hochman S. (2007). Expression and distribution of all dopamine receptor subtypes (D(1)-D(5)) in the mouse lumbar spinal cord: a real-time polymerase chain reaction and non-autoradiographic *in situ* hybridization study. Neuroscience 149, 885–897. 10.1016/j.neuroscience.2007.07.052 17936519 PMC2185067

[B118] Zimmerman A. L. Sawchuk M. Hochman S. (2012). Monoaminergic modulation of spinal viscero-sympathetic function in the neonatal mouse thoracic spinal cord. PLoS ONE 7:e47213. 10.1371/journal.pone.0047213 23144807 PMC3489886

